# Supplementary material for: Predictive value of pulse oximetry for mortality in infants and children presenting to primary care with clinical pneumonia in rural Malawi: A data linkage study
Source: PLoS Med. 2020 Oct 23;17(10):e1003300. doi: 10.1371/journal.pmed.1003300 (PMC7584207; doi:10.1371/journal.pmed.1003300)
Supplement: S1 Appendix — (DOCX) [file pmed.1003300.s002.docx]

**Analysis Plan**

Optimal use of clinical signs for diagnosis and prognosis of childhood pneumonia WHO grant Jan-Jun 2019

**Predictive value of hypoxaemia at community and health centre outpatient levels for mortality in children with pneumonia in Malawi**

V4 Tim Colbourn 30^th^ April 2019

Below we describe the steps of matching the community health worker (CHW) and health centre (HC) outpatient pulse oximetry data to community and inpatient mortality outcomes for childhood pneumonia cases in Lilongwe and Mchinji districts, Malawi from 1^st^ Jan 2012 to 30^th^ June 2014, and determining the association between pulse oximetry measurements (<90% oxygen saturation (SpO2), <93% SpO2, and failed measurement) and 30 day mortality. The final section is on qualitative analysis of verbal autopsy narratives to better understand care seeking pathways.

The PICO for this study is:

**P**opulation: 0-59 month old pneumonia outpatients at CHW and HC levels in Mchinji and Lilongwe districts Malawi.

**I**ntervention (exposure, Tables 1 and 2): Pulse Oximetry at outpatient CHW and HC level for identifying pneumonia patients for referral to higher facilities (hospitals)

**C**omparison (exposure, Tables 1 and 2): use of clinical signs at CHW and HC level for identifying pneumonia patients for referral to higher facilities (hospitals)

**O**utcome: mortality within 30 days of being seen as an outpatient at CHW or HC levels in Lilongwe and Mchinji districts: population mortality from community surveillance in Mchinji district and inpatient mortality from hospitals in Lilongwe and Mchinji districts

1. **Matching**

7,179 pneumonia cases had successful pulse oximetry assessment at CHW level and of these 84 (1.2%) had SpO2 <90%, while 627 (8.7%) had SpO2 between 90 and 92%; an additional 410 had failed measurements at CHW level.[^1^](#_ENREF_1) 6,087 pneumonia cases had successful pulse oximetry assessment at HC level and of these 568 (9.3%) had SpO2 <90%, while 543 (8.9%) had SpO2 between 90 and 92%; an additional 416 had failed measurements.[^1^](#_ENREF_1) Some of these children were referred to hospital for further management and this depended on the presence of chest-indrawing (as per Malawi guidelines), danger signs, and/or SpO2 <90%. Those referred were not tracked in the original study though two other data sources: the routine case report forms for paediatric pneumonia inpatients in hospital[^2^](#_ENREF_2), and population (community) level mortality surveillance data from the VacSurv study (in Mchinji district only)[^3^](#_ENREF_3) gathered information on the outcome of (some of) these cases. The hospital inpatient dataset should contain all those cases successfully referred from CHW and HC levels and recorded as inpatients after arriving at the hospital. Key data on the number of cases in key exposure and outcome categories from each of the four datasets are shown in Table 1.

This is the total available data for this matching and analysis and it is important to note here that not all exposures will be matched to outcomes. Probabilistic matching of the CHW and HC datasets (datasets 1 and 2, Table 1) with the district hospital and population mortality surveillance outcome data (datasets 3 and 4, Table 1) will be undertaken as follows:

Each dataset contains some or all of the following variables: name, care-giver or parent name, age at known date or date of birth, address, ‘sticker number’ (numbered stickers were applied to the health passports of children in the population surveillance) and PCV vaccination dates. For each common variable, approximate matching was used to generate a per-variable score indicating likelihood of match on that variable, for the N1 x N2 pairs of records from the two datasets. A sub-set of data was then examined manually, to identify ‘matches’ and ‘non-matches’. Weights for each variable score were then calculated by performing a regression using the individual variable scores to predict ‘match’ and a combined weighted score calculated using this regression output to determine the most likely matches. The most likely matches were then assessed manually. The manual assessment took into account such factors as different names being spelled in similar ways, the same name being spelled in very different ways, frequency of particular names, different names being used for the same geographical area, and that common mistakes in recording dates may have occurred. In cases of doubt, a second assessor was asked to decide. This process will not detect all true matches, as children who migrate will not match on location and children with changed names will not match on name.

**Table 1: Available data on exposures and outcomes of interest**

| **Dataset** | **Exposures** | **n (%)**  matched deaths (in not referred)  deaths within 30 days (in not referred) | **Outcomes** | **n (%)**  matched deaths  (in referred)  deaths within 30 days  (in referred) |
| --- | --- | --- | --- | --- |
| 1. CHW outpatients (N=7589)^i^ | **a.** SpO2 <90% | 86 (1.1%) 0 0 | Referral decision | 64 (74.4%) 2 1 |
|  | **b.** SpO2 90-92% | 448 (5.9%) 0 0 | Referral decision | 52 (11.6%) 1 1 |
|  | **b2.** SpO2 93%-100% | 6707 (87.6%) 9 3 | Referral decision | 280 (4.2%) 1 1 |
|  | **c.** failed SpO2 measurement | 414 (5.4%) 1 1 | Referral decision | 26 (6.8%) 0 0 |
|  | **d.** Chest-indrawing | 121 (1.5%) 0 0 | Referral decision | 100 (82.6%) 1 1 |
|  | **e.** Danger signs^ii^ (WHO 2014 guidelines clinically eligible for referral) | 879 (11.5%) 0 0 | Referral decision | 93 (10.6%) 1 1 |
|  | **f.** Malawi guidelines clinically eligible for referral = **d. or e.** | 979 (12.8%) 0 0 | Referral decision | 174 (17.8%) 2 2 |
|  | **g.** SpO2 <90% only and NOT WHO clinically eligible = **a & NOT e** | 68 (0.9%) 0 0 | Referral decision | 50 (73.5%) 2 1 |
|  | **h.** SpO2 <90% only and NOT Malawi clinically eligible = **a & NOT f** | 53 (0.7%) 0 0 | Referral decision | 35 (66.0%) 2 1 |
|  | **i.** SpO2 <93% only and NOT WHO clinically eligible = (**a or b) & NOT e** | 454 (5.9%) 0 0 | Referral decision | 88 (19.4%) 3 2 |
|  | **j.** SpO2 <93% only and NOT Malawi clinically eligible = (**a or b) & NOT f** | 423 (5.5%) 0 0 | Referral decision | 59 (14.0%) 2 1 |
|  | **k.** SpO2 <90% and WHO clinically eligible = **a & e** | 18 (0.2%) 0 0 | Referral decision | 14 (77.8%) 0 0 |
|  | **l.** SpO2 <90% and Malawi clinically eligible =  **a & f** | 33 (0.4%) 0 0 | Referral decision | 29 (87.9%) 0 0 |
|  | **m.** SpO2 <93% and WHO clinically eligible = (**a or b) & e** | 80 (1.0%) 0 0 | Referral decision | 28 (35.0%) 0 0 |
|  | **n.** SpO2 <93% and Malawi clinically eligible = (**a or b ) & f** | 111 (1.5%) 0 0 | Referral decision | 57 (51.4%) 1 1 |
|  | **o.** WHO clinically eligible only and SpO2>=90% = **e & NOT (a or c)** | 837 (10.9%) 0 0 | Referral decision | 76 (9.1%) 1 1 |
|  | **p.** Malawi clinically eligible only and SpO2>=90% = **f & NOT (a or c)** | 912 (11.9%) 0 0 | Referral decision | 133 (14.6%) 2 2 |
|  | **q.** WHO clinically eligible only and SpO2>=93% = **e & NOT (a or b or c)** | 775 (10.1%) 0 0 | Referral decision | 62 (8.0%) 1 1 |
|  | **r.** Malawi clinically eligible only and SpO2>=93% = **f & NOT (a or b or c)** | 834 (10.9%) 0 0 | Referral decision | 105 (12.6%) 1 1 |
|  | **s.** failed SpO2 measurement but WHO clinically eligible = **c & e** | 24 (0.3%) 0 0 | Referral decision | 3 (12.5%) 0 0 |
|  | **t.** failed SpO2 measurement but Malawi clinically eligible = **c & f** | 34 (0.4%) 0 0 | Referral decision | 12 (35.3%) 0 0 |
|  | **u.** failed SpO2 measurement and NOT WHO clinically eligible = **c & NOT e** | 390 (5.1%) 1 1 | Referral decision | 23 (5.9%) 0 0 |
|  | **v.** failed SpO2 measurement and NOT Malawi clinically eligible = **c & NOT f** | 380 (5.0%) 1 1 | Referral decision | 14 (3.7%) 0 0 |
|  | **w.** NOT WHO clinically eligible and SpO2>=90% = **NOT e & NOT (a or c)** | 6318 (82.5%) 9 3 | Referral decision | 256 (4.1%) 1 1 |
|  | **x.** NOT WHO clinically eligible and SpO2>=93% = **NOT e & NOT (a or b or c)** | 5932 (77.5%) 9 3 | Referral decision | 218 (3.7%) 0 0 |
|  | **y.** NOT Malawi clinically eligible and SpO2>=90% = **NOT f & NOT (a or c)** | 6243 (81.5%) 9 3 | Referral decision | 199 (3.2%) 0 0 |
|  | **z.** NOT Malawi clinically eligible and SpO2>=93% = **NOT f & NOT (a or b or c)** | 5873 (76.2%) 9 3 | Referral decision | 175 (3.0%) 0 0 |

| 2. HC outpatients (N=6764)^i^ | **a.** SpO2 <90% | 622 (9.2%) 1 1 | Referral decision | 433 (69.6%) 8 5 |
| --- | --- | --- | --- | --- |
|  | **b.** SpO2 90-92% | 576 (8.5%) 1 0 | Referral decision | 231 (41.0%) 3 1 |
|  | **b2.** SpO2 93%-100% | 5114 (75.6%) 11 2 | Referral decision | 695 (13.6%) 5 3 |
|  | **c.** failed SpO2 measurement | 452 (6.7%) 1 1 | Referral decision | 119 (26.3%) 0 0 |
|  | **d.** Chest-indrawing | 1698 (25.1%) 1 1 | Referral decision | 1299 (76.5%) 14 8 |
|  | **e.** Danger signs^ii^ (WHO 2014 guidelines clinically eligible for referral) | 602 (8.9%) 2 1 | Referral decision | 355 (59.0%) 3 3 |
|  | **f.** Malawi guidelines clinically eligible for referral = **d. or e.** | 1942 (28.7%) 2 1 | Referral decision | 1347 (69.4%) 15 9 |
|  | **g.** SpO2 <90% only and NOT WHO clinically eligible **= a & NOT e** | 444 (6.6%) 0 0 | Referral decision | 293 (66.0%) 6 3 |
|  | **h.** SpO2 <90% only and NOT Malawi clinically eligible **= a & NOT f** | 138 (2.0%) 0 0 | Referral decision | 24 (17.4%) 0 0 |
|  | **i.** SpO2 <93% only and NOT WHO clinically eligible **= a or b & NOT e** | 948 (14.0%) 1 0 | Referral decision | 478 (50.4%) 9 4 |
|  | **j.** SpO2 <93% only and NOT Malawi clinically eligible **= a or b & NOT f** | 424 (6.3%) 1 0 | Referral decision | 44 (10.4%) 0 0 |
|  | **k.** SpO2 <90% and WHO clinically eligible **= a & e** | 178 (2.6%) 1 1 | Referral decision | 140 (78.6%) 2 2 |
|  | **l.** SpO2 <90% and Malawi clinically eligible **= a & f** | 484 (7.2%) 1 1 | Referral decision | 409 (84.5%) 8 5 |
|  | **m.** SpO2 <93% and WHO clinically eligible **a or b & e** | 250 (3.7%) 1 1 | Referral decision | 186 (74.4%) 2 2 |
|  | **n.** SpO2 <93% and Malawi clinically eligible **= a or b & f** | 774 (11.4%) 1 1 | Referral decision | 620 (81.0%) 11 6 |
|  | **o.** WHO clinically eligible only and SpO2>=90% = **e & NOT (a or c)** | 363 (5.4%) 1 0 | Referral decision | 177 (48.8%) 1 1 |
|  | **p.** Malawi clinically eligible only and SpO2>=90% = **f & NOT (a or c)** | 1306 (19.3%) 1 0 | Referral decision | 831 (63.6%) 7 4 |
|  | **q.** WHO clinically eligible only and SpO2>=93% = **e & NOT (a or b or c)** | 291 (4.3%) 1 0 | Referral decision | 131 (45.0%) 1 1 |
|  | **r.** Malawi clinically eligible only and SpO2>=93% = **f & NOT (a or b or c)** | 1016 (15.0%) 1 0 | Referral decision | 620 (61.0%) 4 3 |
|  | **s.** failed SpO2 measurement but WHO clinically eligible **= c & e** | 61 (0.9%) 0 0 | Referral decision | 38 (62.3%) 0 0 |
|  | **t.** failed SpO2 measurement but Malawi clinically eligible **= c & f** | 152 (2.3%) 0 0 | Referral decision | 107 (70.4%) 0 0 |
|  | **u.** failed SpO2 measurement and NOT WHO clinically eligible = **c & NOT d** | 391 (5.8%) 1 1 | Referral decision | 81 (20.7%) 0 0 |
|  | **v.** failed SpO2 measurement and NOT Malawi clinically eligible = **c & NOT f** | 300 (4.4%) 1 1 | Referral decision | 12 (4.0%) 0 0 |
|  | **w.** NOT WHO clinically eligible and SpO2>=90% = **NOT e & NOT (a or c)** | 5327 (78.8%) 11 2 | Referral decision | 749 (14.1%) 7 3 |
|  | **x.** NOT WHO clinically eligible and SpO2>=93% = **NOT e & NOT (a or b or c)** | 4823 (71.3%) 10 2 | Referral decision | 564 (11.7%) 4 2 |
|  | **y.** NOT Malawi clinically eligible and SpO2>=90% = **NOT f & NOT (a or c)** | 4384 (64.8%) 11 2 | Referral decision | 95 (2.2%) 1 0 |
|  | **z.** NOT Malawi clinically eligible and SpO2>=93% = **NOT f & NOT (a or b or c)** | 4098 (60.6%) 10 2 | Referral decision | 75 (1.8%) 1 0 |
| 3. Hospital inpatients |  |  | Referred (=N) | 16475 (100.0%) |
|  |  |  | Survived | 15946 (96.8%) |
|  |  |  | Died | 529 (3.2%) |
| 4. Population surveillance |  |  | Total liveborn with verified outcome | 28339 (100.0%) |
|  |  |  | Survived | 26776 (94.5%) |
|  |  |  | Died | 1563 (5.5%) |

^i^ Total N (100%) is exposures w+o+g+k+s+u or y+p+h+l+t+v or x+q+i+m+s+u or z+r+j+n+t+v

^ii^ a composite indicator variable coded as yes (1) if any of the 7 danger signs in Table 2 below are present - this is equivalent to WHO 2014 guidelines clinically eligible for referral. Please note danger signs denoting referral are different for 0<2 month old infants and the danger signs variables will be coded for these very young infants accordingly.

1. **Quantitative Analysis**

The combined dataset of matched records should contain the variables in Table 2 for each pneumonia case. Mortality outcome data will be from inpatient hospital or population surveillance data (datasets 3 and 4, Table 1) and all exposure, confounder and effect modifier variables will be from the outpatient CHW or HC data (datasets 1 and 2, Table 1) except those on socio-economic status variables for the mother which will be from the matched population surveillance data (dataset 4, Table 1).

**Table 2 Variables in combined dataset of pneumonia cases**

| **Variable** | **Unit / Categories (coding)** | **Type**^iii^ **(regression symbol)** |
| --- | --- | --- |
| Mortality^i^ | yes (1), no (0) | Outcome ($Y$) |
| Day of death | days from presentation to CHW of HC to death |  |
| Referred (Hospital inpatient) | yes (1), no (0) | Outcome ($Y_{2}$) |
| Days to referral | days from referral decision to presenting in hospital |  |
| Referred (decision-making indication in outpatient dataset) | yes (1), no (0) | Outcome ($Y_{3}$) |
| SpO2 | % oxygen saturation  / <90%, >=90%, failed measurement (missing)  / <93%, >=93%, failed measurement (missing) | Exposure $X_{1}$ (the intervention):  *<90% threshold model:*  >=90% SpO2: $X_{1\_1}=0$  <90% SpO2: $X_{1\_1}=1$  failed measurement: $X_{1\_1}=2$  *<93% threshold model:*  >=93% SpO2: $X_{1\_2}=0$  <93% SpO2: $X_{1\_2}=1$  failed measurement: $X_{1\_2}=2$ |
| Chest indrawing | yes (1), no (0) | component of Exposure $X_{2\_2}$ |
| Danger signs^ii^ (WHO 2014 guidelines clinically eligible for referral) | yes (1), no (0) | Exposure $X_{2\_1}$ (counterfactual 1) |
| abnormally sleepy | yes (1), no (0) | components of Exposures $X_{2\_1}$ and $X_{2\_2}$ |
| had convulsions | yes (1), no (0) |  |
| not breastfeeding or drinking | yes (1), no (0) |  |
| vomiting everything | yes (1), no (0) |  |
| stridor when calm | yes (1), no (0) |  |
| HIV exposure/infection | yes (1), no (0) |  |
| severe malnutrition | yes (1), no (0) |  |
| Malawi guidelines clinically eligible for referral^iv^ | yes (1), no (0) | Exposure $X_{2\_2}$ (counterfactual 2) |
| CHW/HC level | CHW, HC | Effect Modifier [Strata] |
| Age | months: range 0-59 / 0<2, 2-11, 12-23, 24-59 | Confounder / Effect Modifier |
| Sex | female (1), male (0) | Confounder / Effect Modifier |
| Weight (only available for HC level) | kg / <10, >=10  redo as WAZ | Confounder / Effect Modifier |
| Respiratory Rate | breaths per minute / slow, normal, very fast categories according to age | Effect Modifier |
| Maternal Age ^v^ | years: range 15-49 / 15-19, 20-29, 30-39, 40-49 | Confounder / Effect Modifier |
| Maternal Education ^v^ | none, primary, secondary, tertiary | Confounder / Effect Modifier |
| Maternal Marital Status ^v^ | single, married, previously married (divorced/separated/ widowed) | Confounder / Effect Modifier |
| Wealth index ^v^ (constructed from 8 household items and home construction: Bicycle, oxcart, radio and mobile phone, toilet facility and water source, roof floor and wall material for home construction  Do a Principle Components Analysis to contruct this variable) | five wealth quintiles | Confounder / Effect Modifier |

^i^ within 30 days of being seen at CHW or HC level

^ii^ a composite indicator variable coded as yes (1) if any of the 7 danger signs in the 7 rows below are present - this is equivalent to WHO 2014 guidelines clinically eligible for referral. This is category e. from Table 1 (variable e in dataset). Please note danger signs denoting referral are different for 0<2 month old infants and the danger signs variables will be coded for these very young infants accordingly.

^iii^ Some variables could potentially be confounders or effect modifiers, or exposures, and will be treated as such in iterative and alternative analyses (see below).

^iv^ a composite variable coded as yes (1) if chest indrawing or any of the 7 danger signs in the 7 rows above are present. This is category f. from Table 1 (variable f in dataset)

^v^ Available for those linked with a population record from the population surveillance dataset

**2.1 Descriptive mortality analysis**

We will start by describing the patterns of mortality for each of the exposure categories in Table 1, stratified by whether they were referred or not. This will show the crude differences in mortality rates in those who were documented by the HCW as eligible for referral or not via pulse oximetry, clinical signs, both or neither. As an example, we are interested in any reduction in mortality resulting from HCW identification of those with <90% SpO2 for referral who would not otherwise have been identified without implementation of a pulse oximeter i.e. those who don’t also have clinical signs indicating referral (exposure **g**, Table 1) but are hypoxemic. The difference in mortality rate between the exposure **g** cases who were actually not referred (even though they should have been) compared to those who were referred will indicate the potential net change in mortality from these cases being identified by pulse oximetry and referred.

**Table 3: Descriptive mortality analysis results for CHW and HC data combined**

| **Exposures** | **Not Referred†**  **n**  deaths (%*) | deaths within 30 days (in not referred) (%) | **Referred**  **n**  deaths (%*) | deaths within30 days (in not referred) (%) |
| --- | --- | --- | --- | --- |
| **a.** SpO2 <90% | 197 1 (0.5%) | 1 (0.5%) | 497 10 (2.0%) | 6 (1.2%) |
| **b.** SpO2 90-92% | 403 1 (0.3%) | 0 (0.0%) | 283 4 (1.4%) | 2 (0.7%) |
| **b2.** SpO2 93%-100% | 7155 20 (0.3%) | 5 (0.1%) | 975 6 (0.6%) | 4 (0.4%) |
| **c.** failed SpO2 measurement | 683 2 (0.3%) | 2 (0.3%) | 145 0 (0.0%) | 0 (0.0%) |
| **d.** Chest-indrawing | 407 1 (0.3%) | 1 (0.3%) | 1399 15 (1.1%) | 9 (0.6%) |
| **e.** Danger signs^ii^ (WHO 2014 guidelines clinically eligible for referral) | 431 2 (0.5%) | 1 (0.2%) | 448 4 (0.9%) | 4 (0.9%) |
| **f.** Malawi guidelines clinically eligible for referral = **d. or e.** | 787 2 (0.3%) | 1 (0.1%) | 1521 17 (1.1%) | 11 (0.7%) |
| **g.** SpO2 <90% only and not WHO clinically eligible = **a & NOT e** | 159 0 (0.0%) | 0 (0.0%) | 343 8 (2.3%) | 4 (1.2%) |
| **h.** SpO2 <90% only and not Malawi clinically eligible = **a & NOT f** | 122 0 (0.0%) | 0 (0.0%) | 59 2 (3.4%) | 1 (1.7%) |
| **i.** SpO2 <93% only and not WHO clinically eligible = (**a or b) & NOT e** | 532 1 (0.2%) | 0 (0.0%) | 566 12 (2.1%) | 6 (1.1%) |
| **j.** SpO2 <93% only and not Malawi clinically eligible = (**a or b) & NOT f** | 441 1 (0.2%) | 0 (0.0%) | 103 2 (1.9%) | 1 (1.0%) |
| **k.** SpO2 <90% and WHO clinically eligible = **a & e** | 38 1 (2.6%) | 1 (2.6%) | 154 2 (1.3%) | 2 (1.3%) |
| **l.** SpO2 <90% and Malawi clinically eligible =  **a & f** | 75 1 (1.3%) | 1 (1.3%) | 438 8 (1.8%) | 5 (1.1%) |
| **m.** SpO2 <93% and WHO clinically eligible = (**a or b) & e** | 68 1 (1.5%) | 1 (1.5%) | 214 2 (0.9%) | 2 (0.9%) |
| **n.** SpO2 <93% and Malawi clinically eligible = (**a or b ) & f** | 159 1 (0.6%) | 1 (0.6%) | 677 12 (1.8%) | 7 (1.0%) |
| **o.** WHO clinically eligible only and SpO2>=90% = **e & NOT (a or c)** | 357 1 (0.3%) | 0 (0.0%) | 253 2 (0.8%) | 2 (0.8%) |
| **p.** Malawi clinically eligible only and SpO2>=90% = **f & NOT (a or c)** | 653 1 (0.2%) | 0 (0.0%) | 964 9 (0.9%) | 6 (0.6%) |
| **q.** WHO clinically eligible only and SpO2>=93% = **e & NOT (a or b or c)** | 327 1 (0.3%) | 0 (0.0%) | 193 2 (1.0%) | 2 (1.0%) |
| **r.** Malawi clinically eligible only and SpO2>=93% = **f & NOT (a or b or c)** | 569 1 (0.2%) | 0 (0.0%) | 725 5 (0.7%) | 4 (0.6%) |
| **s.** failed SpO2 measurement but WHO clinically eligible = **c & e** | 36 0 (0.0%) | 0 (0.0%) | 41 0 (0.0%) | 0 (0.0%) |
| **t.** failed SpO2 measurement but Malawi clinically eligible = **c & f** | 59 0 (0.0%) | 0 (0.0%) | 119 0 (0.0%) | 0 (0.0%) |
| **u.** failed SpO2 measurement and not WHO clinically eligible = **c & NOT e** | 647 2 (0.3%) | 2 (0.3%) | 104 0 (0.0%) | 0 (0.0%) |
| **v.** failed SpO2 measurement and not Malawi clinically eligible = **c & NOT f** | 624 2 (0.3%) | 2 (0.3%) | 26 0 (0.0%) | 0 (0.0%) |
| **w.** NOT WHO clinically eligible and SpO2>=90% = **NOT e & NOT (a or c)** | 7201 20 (0.3%) | 5 (0.1%) | 1005 8 (0.8%) | 4 (0.4%) |
| **x.** NOT WHO clinically eligible and SpO2>=93% = **NOT e & NOT (a or b or c)** | 6828 19 (0.3%) | 5 (0.1%) | 782 4 (0.5%) | 2 (0.3%) |
| **y.** NOT Malawi clinically eligible and SpO2>=90% = **NOT f & NOT (a or c)** | 6905 20 (0.3%) | 5 (0.1%) | 294 1 (0.3%) | 0 (0.0%) |
| **z.** NOT Malawi clinically eligible and SpO2>=93% = **NOT f & NOT (a or b or c)** | 6586 19 (0.3%) | 5 (0.1%) | 250 1 (0.4%) | 0 (0.0%) |

† currently doesn’t include those with missing data on referral from CHW data (assume these are not referred?)

* Assuming that no mortality data (i.e. unmatched) are alive

**2.2 Basic regression analysis**

We will then run basic unadjusted logistic regression models to determine the associations between exposure to pulse oximetry oxygen saturation measurement for referral decision-making (the intervention$, X_{1}$), clinical measurements for referral decision-making (the counterfactual$X_{2}$) and mortality (outcome, $Y$). Because the intervention and the counterfactual overlap (many patients were subject to both) they need to be modeled as separate covariates ($X_{1}$ and $X_{2}$), which are both categorical with categories related to thresholds for referral. Adding interaction term ${X_{1}X}_{2}$ enables us to determine how the effect of one changes depending on the presence or absence of the other. The base-case unadjusted model using <90% SpO2 and Malawi guidelines clinical referral criteria thresholds, is:

1. $Y=\ln\left( \frac{\pi_{i}}{1-\pi_{i}} \right)= \beta_{0}+\beta_{1}X_{1\_1}+\beta_{2}X_{2\_1}+{{\beta_{3}X}_{1\_1}X}_{2\_1}+ \varepsilon$

Where $\pi_{i}$ is death for individual *i*, $Y=\ln\left( \frac{\pi_{i}}{1-\pi_{i}} \right)$ is the log odds of death (mortality) i.e. the outcome of interest, the exponent of $\beta_{1}$ is the result of interest: the odds ratio indicating the relative odds of mortality when SpO2 is measured at <90% ($X_{1\_1}$=1) compared to when it is measured at >=90% ($X_{1\_1}$=0; note that those who failed SpO2 measurement will be in a separate third category of this variable $X_{1\_1}$=2 which will have it’s own coefficient in the model relative to $X_{1\_1}$=0, not shown above for simplicity), controlling for decision-making based on presence or absence of Malawi guidelines clinical signs ($X_{2\_1}$). $\beta_{0}$ is the odds of death for an individual at baseline risk (all $X$ variables set to the reference category, in this case of the base-case simple unadjusted model those individuals with SpO2>=90% and who are not clinically eligible for referral according to Malawi guidelines), and $\varepsilon$ is the error term, the residual variation in the outcome $Y$ not explained by the model.

From the above it is apparent that $\beta_{1}$ is the additional effect of use of pulse oximetry on mortality over and above the use of clinical signs. However, because those with SpO2 <90% are sicker and are therefore at a higher risk of mortality we need to consider this by adjusting for the baseline risk of mortality without referral – we need to do this for all exposures in the model (i.e. clinical signs as well) to ensure a fair comparison. We cannot include referral in the regression equation as it is on the causal pathway from exposure to outcome, therefore we need to run the model separately for those who were referred and those who were not. Running the model for those who were referred (mortality with proper referral decision-making) and those who were not (baseline mortality) will enable us to determine the net change in mortality given referral for each intervention / counterfactual exposure group (Table 1). This can be done by using the postestimation **predict xb** command in Stata after each regression is run, summarizing the predicted mortality by exposure group (identified in the dataset via the SpO2 and clinical sign variables) accordingly, and subtracting the predicted mortality for the referred cases from the predicted mortality for the not referred cases for each exposure group. The net change in predicted mortality given identification of those with <90% SpO2 for referral who would not otherwise have been identified i.e. those who don’t also have clinical signs indicating referral, would be that for exposure group **g**, for example. The **predict xb** command uses the regression equation to predict the outcome (mortality) given the covariates. For exposure group **g** where $X_{1\_1}$=1 the **predict xb** command will only utilize coefficient $\beta_{1}$ because $\beta_{2}$ and $\beta_{3}$ will drop out of the equation as $X_{2\_1}$=0, whereas the predicted mortality for exposure group **k** will depend on coefficients $\beta_{1}$, $\beta_{2}$ and $\beta_{3}$ because $X_{1\_1}$=1 and $X_{2\_1}$=1.

We will repeat our analyses changing the pulse oximetry threshold from <90% ($X_{1\_1})$ to <93% ($X_{1\_2})$ and the clinical signs for referral from Malawi ($X_{2\_1})$ to WHO ($X_{2\_2})$guidelines, repeating for each combination of referral thresholds.

Please note that the exposure categories in Table 1 (see footnote i for mutual exclusive category combinations) show the numbers of cases in each matrix ‘cell’ related to the combinations of the covariate categories in the regression model and should provide an indication of the precision of the resulting regression coefficients as well as the tractability of the analysis. There is some concern that some of the categories may be too small especially considering imperfect matching (i.e. some outpatient records not matched to hospital or population mortality data) and small numbers of deaths within each in the final matched dataset (to be determined). To mitigate this in addition to the standard frequentist analysis outlined above we will also do the analysis in a Bayesian framework, specifying uninformative priors for each parameter in the model and estimating the posterior distribution for each given the data – this approach uses the relationships between the parameters specified in the regression model to estimate the effects for each individual and allows extrapolation to the whole population.

**2.3 Primary Analyses (also adjusting for potential confounders)**

We will then run adjusted analyses to control for potential confounding by age, sex, and weight (and for those with data from the population surveillance: maternal age, education, marital status and wealth quintile), by adding these variables to the right hand side of the logistic regression model (1) as additional$X$ variables: first individually in separate regression models, then in combination, and finally all together. We will follow the steps described in $2.2 above to determine predicted net mortality changes given intervention exposures.

**2.4 Clustering**

Some children may have more than one episode of pneumonia in the dataset (we will determine how many via the matching procedures), and clustering of exposures and outcomes is also possible at the CHW, HC or hospital level. We will assess how much clustering there is (how likely the model results are to significantly change due to clustering) and if there is considerable clustering we will adjust for clustering by adding fixed effects by child, and random effects by facility. If this complicates the model to the extend that convergence is an issue we may only do some of these adjustments for clustering.

**2.5 Effect modification**

We will stratify our analyses (§2.2 and §2.3 above) by CHW and HC levels (separate logistic regressions for each level) and also estimate if the association between exposure and mortality differs between CHW and HC level by including CHW/HC level as an effect modifier (interaction term with exposure set $\boldsymbol{X}$) in our main regression model. Given the smaller numbers of cases identified in many of the intervention exposure groups at CHW level (Table 1) it is possible that this analysis will be underpowered and therefore inconclusive.

It is also possible that age, sex, weight (and for those with data from the population surveillance: maternal age, education, marital status and wealth quintile) are effect modifiers rather than confounders, and that respiratory rate is an effect modifier. We will add interaction terms for each of these variables and exposure set $\boldsymbol{X}$ in separate regression equations.

**2.6 Propensity Score Matching and Instrumental Variable Regression**

To improve the robustness of the model to potential confounding we will also explore the use of propensity score matching[^4^](#_ENREF_4) to create exposure groups that are balanced in terms of potential confounding risk factors for referral and mortality (see §2.3 above).

To control for potential unmeasured confounding (confounders not included in the model) we will explore the use of instrumental variable regression – so far we are unable to obtain a suitable instrumental variable i.e. a variable correlated with the exposure but not unobserved characteristics affecting the outcome $\left( \varepsilon\right).$

**2.7 Referral (secondary analysis)**

As a secondary analysis we will determine the association between our exposures and referral to hospital using the same logistic regression equation (1) in §2.2 except substituting the mortality outcome ($Y$) with hospital referral as the outcome ($Y_{2}$ in Table 2). We will also follow steps §2.3–§2.6 for the analysis of this outcome. Like the mortality analysis this analysis assumes those exposures not matched to the hospital inpatient dataset did not go to the hospital. An additional analysis will be done with the referral outcome determined as the referral decision-making indication from the outpatient exposure dataset (outcome $Y_{3}$ in Table 2) and the results compared with the referral outcome based on matched records with the hospital inpatient dataset ($Y_{2})$. The two referral indications will be checked against each other to determine validity.

**2.8 Missing data**

In the basic frequentist regression analysis outlined above we will assume those without data recorded for a yes/no variable are “no” as it is common practice in Malawi to only tick or record positive (i.e. “yes”) information and leave the variable blank if the answer is “no”.

As described at the end of §2.2 we will also do the analysis in a Bayesian framework to impute the missing data via the regression equation. Use of the Bayesian framework could potentially even be used to extrapolate using the matching algorithm to estimate the outcomes for the non-matched (we will need to be careful here) and will also allow extrapolation of the results to the whole population of interest.

**2.9 Sensitivity analyses and Regression Discontinuity analyses**

We will vary the SpO2 threshold to <91%, <92%, <94% and <95% to see if associations with referral and mortality change compared to the <90% and <93% thresholds used.

We will also conduct a regression discontinuity[^5^](#_ENREF_5) analysis to estimate the added benefit of raising the SpO2 threshold i.e. the potential excess mortality averted by such a decision altering referral patterns (and subsequent outcomes) to be similar to those from the use of the <90% rule. This is visually explained in Figure 1 below (note “CFR” should read “mortality”)


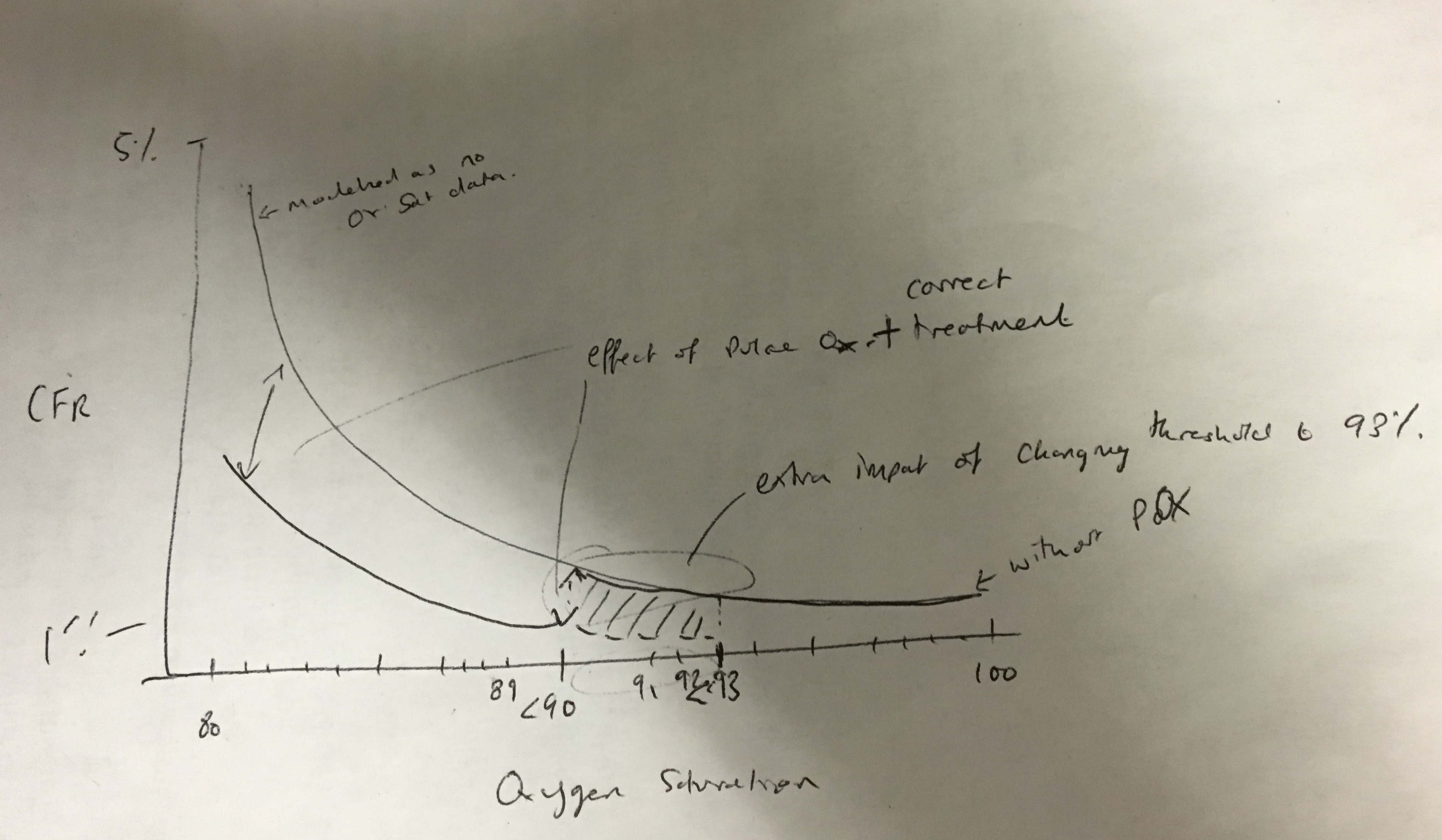


**Figure 1 Representation of mortality reduction given change in referral threshold from <90% SpO2 to <93% as identified via regression discontinuity.** The top line is the counterfactual mortality without pulse oximetry and the bottom line is mortality with pulse oximetry. The bold lines are observed and the dashed and non-bold lines are predicted by regression. The shaded area represents the mortality reduction given the change in referral threshold from <90% SpO2 to <93%.

**2.10 Cost-Effectiveness Analysis and Economic Evaluation**

Using the regression analyses above, we will estimate the cost-effectiveness of the outpatient pulse oximetry intervention in $ per Disability Adjusted Life Years (DALY) averted via the use of the BCEA (Bayesian Cost-Effectiveness Analysis) package in R. This also estimates the probability the intervention will be affordable at different cost-effectiveness (opportunity cost) thresholds as well as the value of information (whether it is worth doing further research) to reduce uncertainty. Tim Colbourn has used this package before in published cost-effectiveness analysis.[^6^](#_ENREF_6)

**3. Qualitative Analysis**

In Mchinji district, all deaths amongst children aged 0-59 months were retrospectively recorded from October 2011 – March 2012, and prospectively recorded from March 2012 to June 2016. All of these deaths were followed-up by a senior monitoring and evaluation officer (MEO), who conducted a standardised 2012 WHO verbal autopsy (VA) closed question interview [see <https://www.who.int/healthinfo/statistics/verbalautopsystandards/en/index2.html>]. In half of these interviews, the interviewer was randomised, at the point of interview, to conduct an open history – a free-text narrative where the respondent tells the story of the child’s death in their own words, with minimal prompting. We will analyse the open narratives of children who died from suspected pneumonia, to determine common patterns in care-seeking behaviour and referrals. Full methods for the process of VA have been previously published.[^7^](#_ENREF_7)

**3.1 Record selection**

During the recruitment period, a total of 4,855 death events were reported, of which 3663 had a VA completed (75%). Reasons for non-completion include migration (9%), no consent (1%) or false reporting of a death event (14%). The following deaths were then excluded from the total sample: stillbirths, deaths over-5 years old, no open history.

From this sample, children who died of a suspected pneumonia were selected. We used two definitions for suspected pneumonia case:

- InterVA-4 [see <http://www.interva.net> ] coded Acute Respiratory Infection or Neonatal Pneumonia as the primary assigned cause of death
- Caregiver responded yes to the presence of an acute cough or difficulty breathing in the 2-weeks prior to death

This resulted in 517 VAs amongst suspected pneumonia deaths, which have an open history, representing 47% of all the suspected pneumonia deaths in the cohort. Table 3 provides a summary of the included records for analysis.

**Table 3 Verbal Autopsy records included in analysis**

| **Variable** | | **N (%)** |
| --- | --- | --- |
| Age | <1 month | 204 (39%) |
|  | 1-12 months | 179 (35%) |
|  | 12-59 months | 134 (26%) |
| Sex | Male | 272 (53%) |
|  | Female | 245 (47%) |
| Travelled to hospital prior to death | Yes | 421 (81%) |
|  | No | 96 (19%) |
| Location of death | Home | 162 (31%) |
|  | Health centre | 126 (24%) |
|  | MDH | 140 (27%) |
|  | On the way to hospital | 33 (6%) |
|  | Other | 56 (11%) |
| Season of death | Rainy | 273 (53%) |
|  | Dry | 244 (47%) |
| Pneumonia classification | InterVA only | 141 (27%) |
|  | Caregiver report only | 211 (41%) |
|  | InterVA + caregiver report | 165 (32%) |

**3.2 Open history translation**

The MEOs were permitted to record the open history in the language they felt more comfortable using at the time of data collection. Of the 517 open histories, 192 (37%) were recorded in Chichewa – the local language in the district. Three of the original MEOs who conducted the VAs were employed to translate these texts from Chichewa to English – discussing any uncertain terms or phrases as a group to come to a consensus.

**3.3 Content analysis**

We will conduct a content analysis of the open histories, with inductive coding allowing the codes to emerge from the data, rather than applying pre-existing theory to the analysis. A random sub-set of 30 interviews will be double coded by TC and CK to establish a common framework of different referral pathways, and the barriers to care-seeking and quality of care at different stages in the referral pathway. The remaining texts will then be coded by CK and an updated coding matrix shared with the study group, and refined through discussion. A random sub-set of 30 texts will then be re-coded by TC to check for consistency and agreement. Any discrepancies will be discussed until a consensus is reached.

The output of this analysis will be description of common patterns in care-seeking and referral, considering delays and barriers at each of these stages. These different pathways will be shared with the MEOs who collected the data, and a sub-set of healthcare providers in Mchinji district to see whether this reflects their understanding and local context. These pathways will be illustrated both visually and with specific case-studies.

**References**

1. McCollum ED, King C, Deula R, et al. Outpatient pulse oximetry implementation with rural facility and community health workers during three years of child pneumonia care in two central Malawi districts. *Bulletin of the World Health Organisation* 2016; **94**: 893-902.

2. McCollum ED, Nambiar B, Deula R, et al. Impact of the 13-valent Pneumococcal Conjugate Vaccine on Clinical and Hypoxemic Childhood Pneumonia over Three Years in Central Malawi: An observational study. *PLoS One* 2017; **DOI:10.1371/journal.pone.0168209 January 4, 2017**.

3. Bar-Zeev N, King C, Phiri T, et al. Impact of monovalent rotavirus vaccine on diarrhoea-associated post-neonatal infant mortality in rural communities in Malawi: a population-based birth cohort study. *The Lancet Global health* 2018; **6**(9): e1036-e44.

4. Wu S, Ding Y, Wu F, Hou J, Mao P. Application of propensity-score matching in four leading medical journals. *Epidemiology* 2015; **26**(2): e19-20.

5. Bor J, Moscoe E, Mutevedzi P, Newell ML, Barnighausen T. Regression discontinuity designs in epidemiology: causal inference without randomized trials. *Epidemiology* 2014; **25**(5): 729-37.

6. Colbourn T, Pulkki-Brännström A-M, Nambiar B, et al. Cost-effectiveness and affordability of community mobilisation through women’s groups and quality improvement in health facilities (MaiKhanda trial) in Malawi. *Cost Effectiveness & Resource Allocation* 2015; **13**(1): 1.

7. King C, Zamawe C, Banda M, et al. The quality and diagnostic value of open narratives in verbal autopsy: a mixed-methods analysis of partnered interviews from Malawi. *BMC Med Res Methodol* 2016; **16**: 13.
