# Supplementary material for: Predictive value of pulse oximetry for mortality in infants and children presenting to primary care with clinical pneumonia in rural Malawi: A data linkage study
Source: PLoS Med. 2020 Oct 23;17(10):e1003300. doi: 10.1371/journal.pmed.1003300 (PMC7584207; doi:10.1371/journal.pmed.1003300)
Supplement: S2 Appendix — (DOCX) [file pmed.1003300.s003.docx]

**S3 Appendix supplementary material**

**Matching**

We used datasets of pneumonia cases at CHW, health centre and hospital levels from Mchinji and Lilongwe districts from January 2012 to June 2014, and community surveillance data from Mchinji district only (see the map in Figure 1 of the paper). The surveillance data, which covered children born on or after 1^st^ Oct 2011, comprised follow-up data collected at 12+ months of age for most children and 4+ months for the rest, and community death reports.

Probabilistic matching of the CHW and HC datasets with the hospital and population mortality surveillance outcome data was done on as many as possible of: name, care-giver or parent name, age at known date or date of birth, address, ‘sticker number’ (numbered stickers were applied to the health passports of children in the population surveillance) and PCV13 vaccination dates. In practice there were few common variables to match on as not all datasets included all these variables. For each common variable, approximate matching was used to generate a per-variable score indicating closeness of match on that variable, for the N1 x N2 pairs of records from the two datasets. A sub-set of data was then examined manually, to identify ‘matches’ and ‘non-matches’. Weights for each variable score were then calculated by performing a regression using the individual variable scores to predict ‘match’. Combined weighted scores were calculated using this regression output to determine the most likely matches. The most likely matches were then assessed manually. The manual assessment took into account such factors as different names being spelled in similar ways, the same name being spelled in very different ways, frequency of particular names, different names being used for the same geographical area, and that common mistakes in recording dates may have occurred. In cases of doubt, a second assessor in Malawi with local geographical knowledge was asked to decide. This process does not detect all true matches, as children who migrate will not match on location and children with changed names will not match on name. Our matching was also limited because the community surveillance mortality data was only collected in Mchinji district, and it only captured children born since October 2011 (i.e., not the older under-5 children recorded as pneumonia cases from January 2012 to June 2014). The recording of geographical areas was also less precise for cases who lived further away from the health facility, as larger geographical units were used to describe such addresses. Some children also remained unmatched to hospital inpatient data because it is known that some children attended hospitals in Zambia and private hospitals from which we did not collect data (see ‘Non-study Facilities’ in map above). Follow-up investigations in Malawi also suggest that some children who we think died of pneumonia in Mchinji (from verbal autopsy data) didn't get diagnosed as pneumonia cases when they presented at the health centre (i.e., were missed cases) so also remain unmatched to our pneumonia outpatient data.

In more detail, for each matching variable, a score from 0 to 1 was calculated, indicating the degree of difference, for each of the N_1_ x N_2_ record pairs. If data were missing, a score of 0.5 was used. For names, each name was regularised, by removing non-alphabetic characters, treating certain letters as equivalent, etc. before comparing the names using strdist, a user-written add-on to Stata, to calculate the cosine string distance between the names. Scores above a cut-off value (0.6) were set to 1 (indicating non-match). For example:

| **Name 1** | **Name 2** | **Score** |
| --- | --- | --- |
| Beatrice Yosephy | Betrice Yolephi | 0.118 |
| yusufu andulu | Yusufu Andrew | 0.200 |
| Vanessa Lester | Vanesa Lesitala | 0.333 |

For ages, the scores were based on the number of months difference in the ages. Other appropriate measures were used for other variables.

These scores were then summed, using the weights in the table below. Sex was treated differently. It was not available in all the datasets, but when present, it was used as a blocking variable (i.e. sex had to agree).

| **Health Centre to Surveillance matching** | | |
| --- | --- | --- |
| Sticker number | 0.386 | Not used for death data |
| Distance from HC | 0.091 |  |
| Village name | 0.282 |  |
| TA name | 0.054 |  |
| Child name | 0.926 |  |
| Child age | 0.327 |  |
|  |  |  |
| **CHW to Surveillance matching** | | |
| Sticker number | 0.165 | Not used for death data |
| CHW catchment area | 0.157 |  |
| Village name | 0.164 |  |
| Child name | 0.612 |  |
| Caregiver name | 0.632 |  |
| Child age | 0.406 |  |
| PCV dates | 0.208 |  |
|  |  |  |
| **Health Centre & CHW to Hospital matching** | | |
| Address | 0.519 |  |
| TA name | 0.266 |  |
| Child name | 1.768 |  |
| Child age | 0.516 |  |
| Visit dates | 0.194 |  |

Please note that the absolute values of the weights are not important, just their relative values within each comparison. Because the outputs of the matching process were then examined manually, it is unlikely that modest changes to the weights would make any difference to the final outcome.

The matching success rates detailed in the paper might be considered low. The table below splits the number of cases by district and by date of birth. Given the community mortality surveillance was only done in Mchinji district and only started in October 2011 we could only match those born from 1^st^ October 2011 in Mchinji district. Therefore a large proportion of under-5 CHW and HC cases (the older ones) and those in Lilongwe are precluded.

|  | **Mchinji** | **Lilongwe** | **Feasible to match** | **Matched (% of feasible)** |
| --- | --- | --- | --- | --- |
| CHW cases | 3,203 | 4,155 |  |  |
| of which DoB≥1Oct2011 | 1,437 | 2,113 | 1437 | 417 (29.0%) |
| Health Centre cases | 4,443 | 2,012 |  |  |
| of which DoB≥1Oct2011 | 2,903 | 1,293 | 2903 | 695 (23.9%) |

So for the HC data instead of the total 6451 cases (also see Table 1 of paper), it was only feasible to match 2903 to the surveillance data, and for 694 of these (23.9%) we have an outcome from the matching.

For the CHW data instead of the total 7362 cases (Table 1 of paper), it was only feasible to match 1437 to the surveillance data, and for 417 of these (29.0%) we have an outcome from the matching.

It was difficult to match data from different levels of the health system (for our referral analysis as well as mortality analysis), as less identifying information was collected at the health centres and hospitals.

**S3 Appendix Tables**

**Table A: Multiple pneumonia episodes in the same child (none of these children died)†**

| **Child** | **Episode** | **CHW or HC** | **Age in months** | **Days since first seen** | **SpO_2_** | **chest indrawing** | **Danger signs**^a^ |
| --- | --- | --- | --- | --- | --- | --- | --- |
| 1 | 1 | CHW | . | Day 0 | 97% | No | No |
|  | 2 | HC | 3 | Day 3 | 94% | No | No |
|  | 3 | CHW | 5 | Day 62 | 88% | Yes | Malawi=WHO |
|  | 4 | HC | 5 | Day 62 | 94% | Yes | Malawi |
|  | 5 | CHW | 6 | Day 95 | 96% | Yes | Malawi=WHO |
|  | 6 | HC | 6 | Day 95 | 97% | No | No |
| 2 | 1 | CHW | . | Day 0 | 97% | No | No |
|  | 2 | HC | 2 | Day 1 | . | Yes | Malawi |
| 3 | 1 | CHW | 20 | Day 0 | 97% | No | No |
|  | 2 | CHW | 21 | Day 0 | . | No | No |
| 4 | 1 | CHW | 15 | Day 0 | . | Yes | Malawi=WHO |
|  | 2 | HC | 15 | Day 0 | 97% | Yes | Malawi |
| 5 | 1 | CHW | 7 | Day 0 | 97% | No | No |
|  | 2 | CHW | 8 | Day 26 | 96% | No | No |
| 6 | 1 | CHW | 12 | Day 0 | 96% | No | No |
|  | 2 | CHW | 13 | Day 23 | 97% | No | No |
| 7 | 1 | CHW | 7 | Day 0 | 93% | Yes | Malawi=WHO |
|  | 2 | HC | 6 | Day 0 | . | Yes | Malawi |
| 8 | 1 | CHW | 5 | Day 0 | . | No | No |
|  | 2 | HC | 5 | Day 0 | 94% | No | No |
|  | 3 | CHW | 6 | Day 28 | . | No | No |
| 9 | 1 | CHW | 15 | Day 0 | 98% | Yes | Malawi=WHO |
|  | 2 | HC | 16 | Day 1 | 98% | No | No |
| 10 | 1 | CHW | . | Day 0 | 97% | No | No |
|  | 2 | CHW | . | Day 28 | 99% | No | No |
| 11 | 1 | CHW | 2 | Day 0 | 96% | No | No |
|  | 2 | CHW | 3 | Day 21 | 97% | No | No |
| 12 | 1 | CHW | 6 | Day 0 | 97% | No | No |
|  | 2 | CHW | 7 | Day 23 | 96% | No | No |
| 13 | 1 | CHW | 10 | Day 0 | 100% | No | No |
|  | 2 | CHW | 7 | Day 0 | 98% | No | No |
| 14 | 1 | CHW | 12 | Day 0 | . | No | No |
|  | 2 | CHW | 12 | Day 1 | . | No | No |
| 15 | 1 | CHW | 5 | Day 0 | 97% | No | No |
|  | 2 | CHW | 6 | Day 12 | 97% | No | No |
| 16 | 1 | CHW | 2 | Day 0 | . | No | No |
|  | 2 | CHW | 5 | Day 27 | . | No | No |
| 17 | 1 | CHW | 11 | Day 0 | . | No | No |
|  | 2 | CHW | 11 | Day 0 | 97% | No | No |
| 18 | 1 | HC | 12 | Day 0 | 98% | No | No |
|  | 2 | HC | 12 | Day 6 | 95% | No | No |
| 19 | 1 | CHW | 2 | Day 0 | 97% | No | No |
|  | 2 | CHW | 2 | Day 9 | 97% | No | No |
| 20 | 1 | CHW | 9 | Day 0 | 91% | Yes | Malawi=WHO |
|  | 2 | HC | 9 | Day 0 | 94% | Yes | Malawi |
| 21 | 1 | HC | 5 | Day 0 | 95% | No | No |
|  | 2 | HC | 7 | Day 20 | . | No | No |
| 22 | 1 | CHW | 5 | Day 0 | 97% | No | No |
|  | 2 | CHW | 6 | Day 28 | 97% | No | No |
|  | 3 | CHW | 7 | Day 41 | 98% | No | No |
| 23 | 1 | HC | 3 | Day 0 | 99% | Yes | Malawi |
|  | 2 | HC | 3 | Day 0 | 99% | Yes | Malawi |
| 24 | 1 | HC | 7 | Day 0 | 97% | No | No |
|  | 2 | CHW | 8 | Day 1 | 96% | No | No |
| 25 | 1 | CHW | 3 | Day 0 | 99% | No | No |
|  | 2 | CHW | 9 | Day 0 | 99% | No | No |
| 26 | 1 | HC | 2 | Day 0 | 99% | No | No |
|  | 2 | CHW | 2 | Day 20 | 96% | No | No |
| 27 | 1 | CHW | 18 | Day 0 | 98% | No | No |
|  | 2 | CHW | 18 | Day 19 | 91% | No | No |
| 28 | 1 | HC | 7 | Day 0 | 91% | Yes | Malawi |
|  | 2 | HC | 7 | Day 0 | 95% | Yes | Malawi |
| 29 | 1 | CHW | 6 | Day 0 | 89% | Yes | Malawi=WHO |
|  | 2 | HC | 6 | Day 0 | 94% | Yes | Malawi |
| 30 | 1 | CHW | 8 | Day 0 | 97% | No | No |
|  | 2 | CHW | 8 | Day 13 | 95% | No | No |
| 31 | 1 | HC | 3 | Day 0 | 97% | Yes | Malawi |
|  | 2 | HC | 4 | Day 25 | 95% | No | No |
| 32 | 1 | HC | 12 | Day 0 | 92% | Yes | Malawi |
|  | 2 | HC | 12 | Day 12 | 96% | Yes | Malawi |

CHW = Community Health Worker HC = Health Centre SpO_2_ = Oxygen saturation

. = missing data (if for SpO_2_ denotes failed SpO_2_ measurement)

†All episodes of pneumonia occurring within 30 days of the previous one are considered multiple episodes.

^a^ see Table 1 of paper for Danger signs: Malawi=WHO: Malawi guidelines are the same as WHO iCCM guidelines for CHW patients. For HC patients Malawi guidelines include chest indrawing as a danger sign, but WHO IMCI guidelines do not. None of the HC episodes had WHO IMCI guideline danger signs.

**Table B Independent associations of SpO_2_ and danger sign exposures on mortality, adjusted logistic regression results**

| **I. Community Health Worker data** | | | | | | | | | | |  |  |
| --- | --- | --- | --- | --- | --- | --- | --- | --- | --- | --- | --- | --- |
| **Model** | | **Coefficient** | | | **Odds Ratio†** | | **95%CI** | | **p-value** | |  |  |
| **M90. Malawi (=WHO) guidelines, <90% SpO_2_ threshold (this was used by the healthcare workers)**  **(N=369)** | | SpO_2_ >=90% | | | 1 (ref) | |  | |  | |  |  |
|  |  | <90% | | | 7.33 | | (0.42, 127.1) | | 0.171 | |  |  |
|  |  | failed | | | 0.41 | | (0.04, 4.52) | | 0.465 | |  |  |
|  |  | Malawi danger signs: absent | | | 1 (ref) | |  | |  | |  |  |
|  |  | : present | | | 2.70 | | (0.46, 15.9) | | 0.272 | |  |  |
|  |  | SpO_2_ <90% X Danger signs | | | (empty)^a^ | | | |  | |  |  |
|  |  | failed SpO_2_ X Danger signs | | | (empty)^a^ | | | |  | |  |  |
|  |  | Age (per month increase) | | | 1.14 | | (1.07, 1.21) | | 0.000**** | |  |  |
|  |  | Sex (female=1 vs. male=0) | | | 0.92 | | (0.30, 2.83) | | 0.883 | |  |  |
|  |  | Respiratory rate^i^: normal | | | (empty – no deaths) | | | |  | |  |  |
|  |  | : fast breathing | | | 1 (ref) | |  | |  | |  |  |
|  |  | : very fast breathing | | | 1.63 | | (0.23, 11.40) | | 0.621 | |  |  |
|  |  | constant (baseline odds) | | | 0.008 | | (0.002, 0.025) | | 0.000 | |  |  |
| **M93. Malawi (=WHO) guidelines, <93% SpO_2_ threshold (N=371)** | | SpO_2_ >=93% | | | 1 (ref) | |  | |  | |  |  |
|  |  | <93% | | | 3.12 | | (0.26, 37.2) | | 0.318 | |  |  |
|  |  | failed | | | 0.39 | | (0.03, 4.47) | | 0.450 | |  |  |
|  |  | Malawi Danger signs: absent | | | 1 (ref) | |  | |  | |  |  |
|  |  | : present | | | 1.37 | | (0.14, 13.0) | | 0.786 | |  |  |
|  |  | SpO_2_ <90% X Danger signs | | | 3.11 | | (0.06, 158.4) | | 0.571 | |  |  |
|  |  | failed SpO_2_ X Danger signs | | | (empty)^b^ | | | |  | |  |  |
|  |  | Age (per month increase) | | | 1.14 | | (1.07, 1.21) | | 0.000**** | |  |  |
|  |  | Sex (female=1 vs. male=0) | | | 1.00 | | (0.32, 3.11) | | 0.997 | |  |  |
|  |  | Respiratory rate^i^: normal | | |  | | | |  | |  |  |
|  |  | : fast breathing | | | 1 (ref) | |  | |  | |  |  |
|  |  | : very fast breathing | | | 1.70 | | (0.25, 11.7) | | 0.789 | |  |  |
|  |  | constant (baseline odds) | | | 0.007 | | (0.002, 0.024) | | 0.000 | |  |  |
| **II. Health Centre data** | | | | | | | | | | | | |
| **Model** |  | | **Age, Sex, Respiratory rate** | | | | | **Age, Sex, Respiratory rate, WAZ** | | | | |
|  | **Coefficient** | | **RR** | **95%CI** | | **p-value** | | **OR**† | | **95%CI** | | **p-value** |
| **M90. Malawi guidelines, <90% SpO_2_ threshold (this was used by the healthcare workers)**  **(left: N=586)**  **(right: N=537**†**)** | SpO_2_ >=90% | | 1 (ref) |  | |  | | 1 (ref) | |  | |  |
|  | <90% | | 3.69 | (1.00, 13.5) | | 0.049 | | 8.89 | | (1.63, 48.4) | | 0.012* |
|  | failed | | 18.1 | (1.72, 190.1) | | 0.016* | | 27.9 | | (2.09, 373) | | 0.012* |
|  | Malawi danger signs: absent | | 1 (ref) |  | |  | | 1 (ref) | |  | |  |
|  | : present | | 3.84 | (0.70, 21.1) | | 0.122 | | 2.53 | | (0.38, 16.8) | | 0.337 |
|  | SpO_2_ <90% X Danger signs | | (empty)^c^ | | |  | |  | | (empty)^c^ | |  |
|  | failed SpO_2_ X Danger signs | | (empty)^c^ | | |  | |  | | (empty)^c^ | |  |
|  | Age (per month increase) | | 1.04 | (0.98, 1.11) | | 0.213 | | 1.05 | | (0.97, 1.13) | | 0.266 |
|  | Sex (female=1 vs. male=0) | | 1.74 | (0.56, 5.41) | | 0.340 | | 3.45 | | (0.76, 15.6) | | 0.108 |
|  | Respiratory rate^i^: normal | | 1 (ref) |  | |  | | 1 (ref) | |  | |  |
|  | : fast breathing | | 0.44 | (0.05, 3.68) | | 0.448 | | 0.35 | | (0.03, 3.85) | | 0.392 |
|  | : very fast breathing | | 1.23 | (0.14, 10.9) | | 0.852 | | 0.59 | | (0.04, 8.32) | | 0.694 |
|  | WAZ^ii^  normal (> -2 z-scores) | |  |  | |  | | 1 (ref) | |  | |  |
|  | low (-3 > -2 z-scores) | |  |  | |  | | (empty)^c^ | | | |  |
|  | severely low (< -3 z-scores) | |  |  | |  | | 19.5 | | (2.96, 128) | | 0.002** |
|  | constant (baseline odds) | | 0.006 | (0.0005, 0.07) | | 0.000 | | 0.004 | | (0.0002, 0.06) | | 0.000 |

| **Model** |  | **Age, Sex, Respiratory rate** | | | | **Age, Sex, Respiratory rate, WAZ** | | |  |
| --- | --- | --- | --- | --- | --- | --- | --- | --- | --- |
|  | **Coefficient** | **RR** | **95%CI** | | **p-value** | **RR** | **95%CI** | **p-value** |  |
| **M93. Malawi guidelines, <93% SpO_2_ threshold (left: N=565)**  **(right: N=519)** | SpO_2_ >=93% | 1 (ref) |  | |  | 1 (ref) |  |  |  |
|  | <93% | 2.77 | (0.70, 10.9) | | 0.145 | 3.65 | (0.76, 17.6) | 0.107 |  |
|  | failed | 17.2 | (1.64, 180.6) | | 0.018* | 20.8 | (1.93, 225) | 0.012* |  |
|  | Malawi Danger signs: absent | 1 (ref) |  | |  | 1 (ref) |  |  |  |
|  | : present | 3.38 | (0.56, 20.3) | | 0.184 | 2.43 | (0.35, 16.9) | 0.369 |  |
|  | SpO_2_ <90% X Danger signs | (empty)^c^ | | |  |  | (empty)^c^ |  |  |
|  | failed SpO_2_ X Danger signs | (empty)^c^ | | |  |  | (empty)^c^ |  |  |
|  | Age (per month increase) | 1.04 | (0.98, 1.11) | | 0.193 | 1.03 | (0.95, 1.11) | 0.481 |  |
|  | Sex (female=1 vs. male=0) | 1.82 | (0.57, 5.77) | | 0.309 | 2.16 | (0.67, 6.91) | 0.196 |  |
|  | Respiratory rate^i^: normal | 1 (ref) |  | |  | 1 (ref) |  |  |  |
|  | : fast breathing | 0.42 | (0.05, 3.61) | | 0.427 | 0.48 | (0.06, 3.76) | 0.482 |  |
|  | : very fast breathing | 1.32 | (0.15, 12.0) | | 0.803 | 1.70 | (0.18, 16.0) | 0.641 |  |
|  | WAZ^ii^  normal (> -2 z-scores) |  |  | |  | 1 (ref) |  |  |  |
|  | low (-3 > -2 z-scores) |  |  | |  | (empty)^c^ | |  |  |
|  | severely low (< -3 z-scores) |  |  | |  | 6.17 | (1.36, 27.9) | 0.018* |  |
|  | constant (baseline odds) | 0.006 | (0.0005, 0.07) | | 0.000 | 0.005 | (0.0004, 0.05) | 0.000 |  |
| **W90. WHO guidelines, <90% SpO_2_ threshold (left: N=611)**  **(right: N=560)** | SpO_2_ >=90% | 1 (ref) |  | |  | 1 (ref) |  |  |  |
|  | <90% | 7.07 | (1.59, 31.4) | | 0.010* | 7.59 | (1.77, 32.6) | 0.006** |  |
|  | failed | 4.74 | (0.56, 40.5) | | 0.155 | 5.91 | (0.67, 52.3) | 0.110 |  |
|  | WHO Danger signs: absent | 1 (ref) |  | |  | 1 (ref) |  |  |  |
|  | : present | 3.83 | (0.71, 20.6) | | 0.117 | 1.56 | (0.17, 14.2) | 0.693 |  |
|  | SpO_2_ <90% X Danger signs | 0.34 | | (0.03, 3.64) | 0.374 | 1.06 | (0.06, 17.9) | 0.967 |  |
|  | failed SpO_2_ X Danger signs | (empty)^d^ | | |  |  | (empty)^d^ |  |  |
|  | Age (per month increase) | 1.05 | (0.98, 1.12) | | 0170 | 1.06 | (1.00, 1.13) | 0.066 |  |
|  | Sex (female=1 vs. male=0) | 1.86 | (0.59, 5.90) | | 0.290 | 2.93 | (0.82, 10.5) | 0.099 |  |
|  | Respiratory rate^i^: normal | 1 (ref) |  | |  | 1 (ref) |  |  |  |
|  | : fast breathing | 0.40 | (0.05, 3.32) | | 0.393 | 0.24 | (0.03, 2.23) | 0.211 |  |
|  | : very fast breathing | 1.07 | (0.11, 10.1) | | 0.953 | 0.50 | (0.05, 5.54) | 0.572 |  |
|  | WAZ^ii^  normal (> -2 z-scores) |  |  | |  | 1 (ref) |  |  |  |
|  | low (-3 > -2 z-scores) |  |  | |  | (empty)^e^ | |  |  |
|  | severely low (< -3 z-scores) |  |  | |  | 8.63 | (2.27, 32.9) | 0.002** |  |
|  | constant (baseline odds) | 0.008 | (0.001, 0.08) | | 0.000 | 0.007 | (0.0007, 0.08) | 0.000 |  |
| **W93. WHO guidelines, <93% SpO_2_ threshold (left: N=611)**  **(right: N=560)** | SpO_2_ >=93% | 1 (ref) |  | |  | 1 (ref) |  |  | |
|  | <93% | 5.16 | (1.14, 23.3) | | 0.033* | 4.76 | (1.07, 21.2) | 0.040* | |
|  | failed | 5.53 | (0.60, 50.7) | | 0.131 | 6.09 | (0.66, 55.8) | 0.110 | |
|  | WHO Danger signs: absent | 1 (ref) |  | |  | 1 (ref) |  |  | |
|  | : present | 5.32 | (0.91, 31.0) | | 0.063 | 2.56 | (0.28, 23.2) | 0.405 | |
|  | SpO_2_ <93% X Danger signs | 0.29 | | (0.03, 3.29) | 0.320 | 0.62 | (0.04, 10.5) | 0.743 | |
|  | failed SpO_2_ X Danger signs | (empty)^d^ | | |  |  | (empty)^d^ |  | |
|  | Age (per month increase) | 1.04 | (0.98, 1.11) | | 0.202 | 1.04 | (0.97, 1.11) | 0.265 | |
|  | Sex (female=1 vs. male=0) | 1.89 | (0.58, 6.15) | | 0.292 | 2.37 | (0.69, 8.14) | 0.172 | |
|  | Respiratory rate^i^: normal | 1 (ref) |  | |  | 1 (ref) |  |  | |
|  | : fast breathing | 0.42 | (0.05, 3.68) | | 0.433 | 0.50 | (0.05, 4.82) | 0.551 | |
|  | : very fast breathing | 1.25 | (0.13, 12.3) | | 0.847 | 1.35 | (0.12, 14.6) | 0.806 | |
|  | WAZ^ii^  normal (> -2 z-scores) |  |  | |  | 1 (ref) |  |  | |
|  | low (-3 > -2 z-scores) |  |  | |  | (empty)^e^ | |  | |
|  | severely low (< -3 z-scores) |  |  | |  | 5.92 | (1.46, 24.0) | 0.013* | |
|  | constant (baseline odds) | 0.007 | (0.0007, 0.07) | | 0.000 | 0.005 | (0.0004, 0.06) | 0.000 | |

OR = Odds Ratio RR = Risk Ratio (ref) = Reference (baseline) category

Statistically significant differences are denoted: * p<0.05; ** p<0.01; *** p<0.001; **** p<0.0001

† The CHW general linear models with binomial family and log link (GLM) did not converge with Age added (and neither did the HC M90, Age, sex,, respiratory rate, WAZ model), therefore we report the analagous logistic regression models for these adjusted analyses. These models report results in Odds Ratios (OR) rather than Risk Ratios (RR). The Odds Ratios are more extreme than the risk ratios, though not by much given death is a relatively rare outcome (16/369=4.3%) – as an example in the adjusted model with sex only the RR for the SpO_2_<90% coefficient is 6.87 and the OR is 8.63. Please note the SpO_2_<90% coefficient for the unadjusted logistic regression model is not p<0.05 significant [OR: 8.81 (95%CI: 0.85, 91.0), p=0.068] but in the GLM it is p<0.05 significant [RR: 6.85 (95%CI: 1.15, 40.9), p=0.035, Table 2 of paper]

X = interaction term. Please note that we know these models are correctly specified because they predict the observed mortality rates for each category shown in Table 2.

(empty) = no deaths in this group so coefficient not possible to estimate

^i^ fast breathing: >=60 and <=79, >=50 and <=69, >=40 and <=59 breaths per minute for <2, 2-11 and 12-59 months of age categories; very fast breathing: >=80, >=70, >=60 breaths per minute for <2, 2-11 and 12-59 months of age categories. ‘fast breathing’ is the reference category in the CHW models because there were no deaths in the ‘normal’ respiratory rate group. In the HC data There was only one death in the ‘normal’ respiratory rate group (the reference category).

^ii^ Weight for Age Z-Score (WAZ) calculated from age in months and weight in kilograms, using WHO growth curves for males and females separately via the zanthro command in Stata

^a^ see Table 2A of paper, top left orange panel and top right yellow panel n=3 and 0 deaths in group “SpO_2_ <90% but Malawi clinically eligible” and n=5 and 0 deaths in group “failed SpO_2_ measurement but Malawi clinically eligible”

(empty) = no deaths in this group so coefficient not possible to estimate

^b^ see Table 2A of paper, top left orange panel and top right yellow panel n=5 and 0 deaths in group “failed SpO_2_ measurement but Malawi clinically eligible”

^c^ see Table B top left orange panel and top right yellow panel n=34 and 0 deaths in group “failed SpO_2_ measurement but Malawi clinically eligible” and n=15 (SpO_2_<90%) or n=39 (SpO_2_<93%) and 0 deaths in group “SpO_2_ <90% (<93%) only and not Malawi clinically eligible”

^d^ see Table B bottom left green panel and bottom right blue panel n=20 and 0 deaths in group “failed SpO_2_ measurement but WHO clinically eligible”

^e^ There were no deaths in this WAZ category

The CHW only adjusted models (Table B.I) are qualitatively similar to the CHW unadjusted models. The coefficient for age was significant in both CHW adjusted models (Table B.I) and indicated an increase in odds of mortality in older children as indicated by the mean age of the 16 deaths being 20 months and the mean age of the 385 cases who survived with age data being 9 months (t-test p-value 0.000, data not shown). This may be an artefact of the sample of matched cases being younger than the sample of unmatched cases (Table 1) and the deaths within 30 days being more likely to have been identified (matched) than those who survived up to 30 days post-diagnosis, i.e., we may have picked up more of the deaths in older children than deaths in younger children (it’s possible that children’s names become more ‘stable’ and easier to track the older they get). The coefficients for sex and respiratory rate (‘fast breathing’ and ‘very fast breathing’ relative to ‘normal’ respiratory rate) were not significant either of the CHW adjusted models (Table B.I). Note the ‘normal’ breathing category only had one death, which had low SpO_2_ (83%) and danger signs and died in hospital the same day (this case was from the HC data).

The results of the HC only adjusted models (Table B.II) are also qualitatively similar to the unadjusted HC models. Sex and respiratory rate (‘fast breathing’ and ‘very fast breathing’ relative to ‘normal’ respiratory rate) are not significantly associated with death in these models. Note the ‘normal’ breathing category only had one death in the HC data, which had low SpO_2_ (83%) and danger signs and died in hospital the same day. Age is also not significant in the HC adjusted models as although the 13 deaths are slightly older (mean age 10.7 months, SD: 6.5 months) than the 682 cases who survive (mean age 8.2 months, SD: 6.5 months) this difference is not statistically significant (t-test: p=0.090, data not shown).

Weight and hence Weight for Age Z-score (WAZ) was only available for the HC cases. WAZ <-3 Z-scores (very severe underweight for age) is significantly associated with death in all models (right hand side of S1 Appendix Table B.II) as expected (There were 3 deaths out of the 28 cases in this category (10.7%)). There were no deaths in the low WAZ category (-3 to -2 Z-scores), which consequently drops out of the models (right hand side of S1 Appendix Table B.II; there were 8/561=1.4% deaths in the normal WAZ >-2 Z-score reference category). The fully adjusted HC only models with age, sex, respiratory rate and WAZ included as well as the SpO_2_ and danger signs exposure combinations from the unadjusted models have too many variables in them considering there are only 13 deaths in the HC dataset – this is apparent by the coefficients being very unstable (very wide confidence intervals, right hand columns, S1 Appendix Table B.II), even just compared to the adjusted model with age, sex and respiratory rate included, which already have very wide confidence intervals (middle columns, S1 Appendix Table B.II). This is also the case with the CHW adjusted models compared to the unadjusted models, and as such, given that the coefficients for the SpO_2_ and danger signs variables (i.e. our results of interest) do not qualitatively change between the unadjusted and adjusted models, we prefer the more stable unadjusted models (Table 3 of paper).

**Further detail on referral results:**

Table 5 of the paper shows the results of our regression analyses of the independent associations of SpO_2_ and danger sign exposures on outpatient referral decisions and, separately, hospital referrals (hospital inpatient within 7 days of outpatient diagnosis), for the CHW (Table 5A) and HC (Table 5B) data. These models predict the observed referral rates for each category shown in Table 4 of the paper. Both SpO_2_ threshold eligibility and clinical danger sign referral eligibility were found to be strongly associated with an outpatient referral decision (middle columns of Table 5) as expected, and Malawi clinical danger signs were particularly associated with a referral decision, which is also as expected given the healthcare workers were supposed to be following Malawi guidelines. In the WHO guideline scenarios for HC cases (Models W90 and W93, Table 5B) SpO_2_ thresholds were relatively more associated with outpatient referral decisions than clinical danger signs compared to in the Malawi guideline scenarios (Models M90 and M93, Table 5B) also probably because the healthcare workers were following the Malawi guidelines, which include chest indrawing and capture more of the SpO_2_<90% and SpO_2_<93% cases (Table 2). Failed SpO_2_ measurement was also independently associated with an outpatient referral decision in the WHO guideline scenarios but not the Malawi guideline scenarios (also reflecting the differences in the data shown in Table 4).

The findings for the models of hospital referral (right hand columns of Table 5B) are similar to those of the models of outpatient referral decision (middle columns of Table 5B) though the independent associations of SpO_2_ threshold and clinical danger signs with hospital referral are less strong (risk ratios are not as high) and there are no significant independent associations between failed SpO_2_ measurement and hospital referral.

Hospital referrals were significantly associated with mortality: 12 (17%) of the 70 HC episodes who became hospital inpatients within 7 days of outpatient diagnosis died compared to 1 (0.2%) of the 625 episodes who we did not find to be hospital inpatients (chi-squared test: p=0.000). HC outpatient episodes with referral decision indications were also more likely to die: 9 (4.3%) out of 211, than those who did not have an outpatient referral decision indication: 4 (0.8%) out of 480 (chi-squared test: p=0.002). Hospital referrals had higher deaths rates amongst those without outpatient referral decisions [4/10 (40%) hospital referrals vs. 0/474 (0%) not hospital referrals, chi-squared test: p=0.000] as well as amongst those with outpatient referral decisions [8/60 (13%) hospital referrals vs. 1/151 (0.7%) not hospital referrals, chi-squared test: p=0.000].

**Discussion of not hypoxaemic or clinically eligible for referral deaths**

It is important to note that 14 (48%) of the 29 deaths we found within seven days of diagnosis at CHW and HC levels combined were not hypoxemic or clinically eligible for referral highlighting the importance of follow-up and continuing case management, as well as more specific diagnostic approaches. 12 of these 14 were CHW cases meaning that the 12 cases represent 12/417=2.9% of all CHW cases, and only 2/695=0.3% of HC cases. Clinical signs may have been missed in some of these cases, suggesting improvements in CHW training may be required. It is also possible that many of these cases deteriorated after CHW diagnosis and that mothers are more likely to bring sicker kids straight to the HC rather than the CHW (Table A showing multiple episodes of care-seeking in the same child suggests this). Although all 14 of these deaths were not referred by the outpatient healthcare worker, 2 of them were found to have become hospital inpatients and died in hospital 3 and 4 days after outpatient diagnosis, presumably brought to the hospital by their caregivers following deterioration. Interestingly both of these 2 were the only two following HC diagnosis not among the 12 following CHW diagnosis, suggesting they may already have been sicker, brought straight to a HC where their signs of sickness were missed, and then brought to the hospital a few days later and died there. From our forthcoming analysis of the verbal autopsies of pneumonia deaths (personal communication C. King, February 2020), 40% of children sought care more than once before dying, and many had had several previous episodes of illness before the one that killed them.
