# Supplementary material for: Predictive value of pulse oximetry for mortality in infants and children presenting to primary care with clinical pneumonia in rural Malawi: A data linkage study
Source: PLoS Med. 2020 Oct 23;17(10):e1003300. doi: 10.1371/journal.pmed.1003300 (PMC7584207; doi:10.1371/journal.pmed.1003300)
Supplement: Attachment — Submitted filename: Response_to_reviewers_Outpatient pulse oximetry mortality.docx [file pmed.1003300.s004.docx]

-----------------------------------------------------------
Requests from the editors:

1. Title: Please revise your title according to PLOS Medicine's style. Your title must be nondeclarative and not a question. It should begin with main concept if possible. "Effect of" should be used only if causality can be inferred, i.e., for an RCT. Please place the study design ("A randomized controlled trial," "A retrospective study," "A modelling study," etc.) in the subtitle (ie, after a colon).

We have revised the title by adding “: a data linkage study” to the end. We could alternatively add more detail such as “: a data linkage study of prospective health facility and community case and mortality data” though worry the title is already very long. We believe the rest of the title is fine though are happy to change it at your specific request. Thank you.

2. Abstract- Please structure your abstract using the PLOS Medicine headings (Background, Methods and Findings, Conclusions). Funding is not required here. 
Abstract methods and findings- please provide demographics, places where this study took place in Malawi along with dates
Abstract Methods and Findings:

We have made the required changes. We have deleted the funding section of the abstract. We have added places in Malawi and dates to methods section of abstract as a new second sentence: “*All data was collected in Lilongwe and Mchinji districts, Malawi, from January 2012 to June 2014*”. For the demographics we already say “*children 0-59 months old*” and do not provide any further demographic details in the paper.

3. * Please ensure that all numbers presented in the abstract are present and identical to numbers presented in the main manuscript text.

Thanks, we have checked and whilst all the numbers are in the tables we have now added *“(RR: 9·37, (95% CI: 2·17, 40·4))*” to line 276 of the main text. All other numbers in the abstract are in the main text as well as the tables.

4. * Please include the study design, population and setting, number of participants, years during which the study took place, length of follow up, and main outcome measures.

We have added all these details to the Abstract methods section:
**Study design:** “We conducted a data linkage study of prospective health facility and community case and mortality data.”

**Population**: “From 7358 CHW and 6546 HC pneumonia episodes…”

**Setting:** already added – see point 2 above, and community health worker, health centre and outpatient and community data already mentioned.

**Number of participants:** “…we linked 417 CHW and 695 HC pneumonia episodes to 30-day mortality outcomes; 16 (3.8%) CHW, and 13 (1.9%) HC episodes died.”

**Years during which the study took place:** this has been added in response to point 2 above.

**Length of follow-up:** this is already included in line 39 “including episodes followed up to, and deaths within, 30 days of pneumonia diagnosis”

**Main outcome measures:** this is already included in the background section of the abstract: “We sought to determine mortality outcomes”

5. * Please quantify the main results (with 95% CIs and p values).

The main results already have 95%CIs, we have added the p-values

6. * Please include the important dependent variables that are adjusted for in the analyses.

We have added: “(for age, sex, respiratory rate and, in analyses of HC data only Weight for Age Z-score (WAZ)” after “adjusted” in the methods section of the abstract. Although this sentence is now long with this added in brackets, we’ve done it this way to minimize the total number of words in the abstract.

7. Abstract methods and findings- the last sentence  should include a limitation of your study design

We have added: “The main limitation of our study was our ability to only match 6% of CHW episodes and 11% of HC episodes to mortality outcome data.” as the last sentence of the Methods and Findings section of our abstract.

8. Abstract conclusions- * Please address the study implications without overreaching what can be concluded from the data; the phrase "In this study, we observed ..." may be useful. * Please interpret the study based on the results presented in the abstract, emphasizing what is new without overstating your conclusions. * Please avoid vague statements such as "these results have major implications for policy/clinical care". Mention only specific implications substantiated by the results. * Please avoid assertions of primacy ("We report for the first time....")

Thanks, we believe our conclusion already succeeds on these counts: “Pulse oximetry identified fatal pneumonia episodes at HCs in Malawi that would otherwise have been missed by WHO referral guidelines alone. Our data indicates that chest indrawing should be restored as an indicator for referral to WHO guidelines in high mortality settings.”

9. Please remove the “research in context” section. At this stage, we ask that you include a short, non-technical Author Summary of your research to make findings accessible to a wide audience that includes both scientists and non-scientists. The Author Summary should immediately follow the Abstract in your revised manuscript. This text is subject to editorial change and should be distinct from the scientific abstract.

Please see our author guidelines for more information: <https://journals.plos.org/plosmedicine/s/revising-your-manuscript#loc-author-summary>.

We have replaced the ‘research in context’ section with an Author Summary, following your guidelines, as requested:

**Author Summary**

**Why was this study done?**

- Pneumonia is a leading cause of death of children under 5 years old and early identification and treatment of severe cases is required to prevent deaths
- Pulse oximetry is more sensitive at detecting hypoxaemia than clinical signs alone and therefore can potentially prevent more deaths from pneumonia
- There is a lack of evidence of the effect on child deaths of pulse oximetry use by healthcare workers in informal community settings and at formal primary care clinics and this study sought to fill this evidence gap

**What did the researchers do and find?**

- We linked Malawian community health worker and health centre outpatient data to hospital and community mortality data to determine the mortality outcomes for children with pneumonia identified by pulse oximetry or clinical signs or both as outpatients
- We show that pulse oximetry identified fatal episodes of childhood pneumonia that did not have identified clinical signs
- Including failed attempted pulse oximetry and pulse oximetry readings of less than 90% oxygen saturation identified 12.5% of deaths at community health worker level (2/16) and 31% of deaths at health centre level (4/13) not identified by clinical signs
- Increasing the threshold to less than 93% oxygen saturation, pulse oximetry identified one additional death (5/13, 38% of deaths) not identified by clinical signs at health centre level only
- All of the health centre deaths identified by pulse oximetry – except the one identified by failed attempted pulse oximetry ­– were also identified by chest indrawing in this high-mortality setting.

**What do these findings mean?**

- Pulse oximetry should be used during outpatient community health worker and health centre management of infants and children with suspected pneumonia.
- A SpO_2_ <93% –and failure to obtain a SpO_2_ reading– should be considered as indications for referral depending upon local health system capacities, especially accounting for human resources and oxygen delivery capabilities.
- In the absence of pulse oximetry, chest-indrawing alone should be included as a criterion requiring hospital referral in high infant and child mortality low- and middle-income countries at health centre level.

10. The Data Availability Statement (DAS) requires revision. For each data source used in your study: 

a) If the data are freely or publicly available, note this and state the location of the data: within the paper, in Supporting Information files, or in a public repository (include the DOI or accession number).

b) If the data are owned by a third party but freely available upon request, please note this and state the owner of the data set and contact information for data requests (web or email address). Note that a study author cannot be the contact person for the data.

c) If the data are not freely available, please describe briefly the ethical, legal, or contractual restriction that prevents you from sharing it. Please also include an appropriate contact (web or email address) for inquiries (again, this cannot be a study author).

We are unable to make the original data available as it contains personally identifiable information, which is key to the matching process. Our data linkage study uses data from three publications (references [6], [13] and [14] of the paper):

6.	McCollum ED, King C, Deula R, Zadutsa B, Mankhambo L, Nambiar B, et al. Outpatient pulse oximetry implementation with rural facility and community health workers during three years of child pneumonia care in two central Malawi districts. Bulletin of the World Health Organisation. 2016;94:893-902.

13.	McCollum ED, Nambiar B, Deula R, Zadutsa B, Bondo A, King C, et al. Impact of the 13-valent Pneumococcal Conjugate Vaccine on Clinical and Hypoxemic Childhood Pneumonia over Three Years in Central Malawi: An observational study. PLoS One. 2017;DOI:10.1371/journal.pone.0168209 January 4, 2017.

14.	Bar-Zeev N, King C, Phiri T, Beard J, Mvula H, Crampin AC, et al. Impact of monovalent rotavirus vaccine on diarrhoea-associated post-neonatal infant mortality in rural communities in Malawi: a population-based birth cohort study. The Lancet Global health.

We can make available the matched/unmatched data actually used for the analysis (just the relevant variables, with personal identifiable information removed) and could upload that to a free to access public repository such as the one held by the corresponding authors university UCL. Please let us know if this is acceptable.

11. Square brackets placement- please add a space between text and square brackets followed by a full stop. For example: xxxxxx [3-5].

Done

12. Did your study have a prospective protocol or analysis plan? Please state this (either way) early in the Methods section. a) If a prospective analysis plan (from your funding proposal, IRB or other ethics committee submission, study protocol, or other planning document written before analyzing the data) was used in designing the study, please include the relevant prospectively written document with your revised manuscript as a Supporting Information file to be published alongside your study, and cite it in the Methods section. A legend for this file should be included at the end of your manuscript. b) If no such document exists, please make sure that the Methods section transparently describes when analyses were planned, and when/why any data-driven changes to analyses took place. c) In either case, changes in the analysis-- including those made in response to peer review comments-- should be identified as such in the Methods section of the paper, with rationale.

We have added our pre-specified analysis plan as supplementary material S1 Appendix (the previous S1 Appendix now becomes S3 Appendix as we also add the STROBE checklist as S2 Appendix) and have referenced it where we already mention it at the end of the first paragraph of the methods section: “We developed and then followed our pre-specified analysis plan (S1 Appendix) as far as we were able given the limitations of the final matched dataset”. We add here an additional sentence: “We added the sensitivity and specificity analyses at the request of the statistical reviewer.” These are described at the end of the Analysis sub-section of the Methods: “We calculated the sensitivity and specificity of clinical and SpO2 eligibility on the mortality outcome and compared it to the sensitivity and specificity of clinical eligibility only, for SpO2 eligibility thresholds of <90% and <93% SpO2, WHO and Malawi eligibilities, at CHW and HC levels. We included SpO2 failed measurements, as well as SpO2 below threshold as SpO2 eligible in these analyses, given failed SpO2 measurements are associated with mortality.”

In paragraphs 4 and 5 of the Analysis sub-section of our Methods section we describe planned analyses we were unable to conduct and why we were unable to conduct them (this section was included previously and we have added the sentence on regression discontinuity and clarified the planned effect modification analyses, changes in bold and in tracked changes in the revised manuscript):

“Too few deaths in each exposure group prevented assessment of effect modification by age group, sex **or CHW or HC level**. Although we adjusted for clustering of illness episodes by child and CHW and HC catchment area these models were unstable and not presented. Our unadjusted models were robust to clustering, with similar headline results following 100 iterations for models that did not converge.

The extent of missing data on outcomes due to the majority of outpatient episodes remaining unmatched to 30-day post-diagnosis survival (mortality) outcomes (Table 1) meant that multiple imputation of the missing outcome data was not feasible. **The small numbers of deaths and episodes with low SpO2 also precluded our planned regression discontinuity analyses of the effect of changing the SpO2 threshold on mortality and referral outcomes.**”

13. Please ensure that the study is reported according to the STROBE guideline, and include the completed STROBE checklist as Supporting Information. Please add the following statement, or similar, to the Methods: "This study is reported as per the Strengthening the Reporting of Observational Studies in Epidemiology (STROBE) guideline (S1 Checklist)." The STROBE guideline can be found here: <http://www.equator-network.org/reporting-guidelines/strobe/> When completing the checklist, please use section and paragraph numbers, rather than page numbers.

We have completed the STROBE checklist and included it in our submission as S2. We have added the following sentence to the end of the first paragraph of our Methods section: “This study is reported as per the Strengthening the Reporting of Observational Studies in Epidemiology (STROBE) guideline (S2 Checklist).”

14. For all observational studies, in the manuscript text, please indicate: (1) the specific hypotheses you intended to test, (2) the analytical methods by which you planned to test them, (3) the analyses you actually performed, and (4) when reported analyses differ from those that were planned, transparent explanations for differences that affect the reliability of the study's results. If a reported analysis was performed based on an interesting but unanticipated pattern in the data, please be clear that the analysis was data-driven.

Please see our response to your point 12 above, we believe we now do this.

15. Role of funding source should be added into the financial statement within the article metadata and removed from the main text

We have removed the Role of funding source section from the main text, and it is now only in the metadata included with our online submission.

16. Please conclude the Introduction with a clear description of the study question or hypothesis.

We have added this to the end of our introduction section: “We hypothesized that pulse oximetry use for outpatients could identify children at risk of dying who would not be identified by clinical signs alone.”

17. Conclusions must be toned down since this is an observational study

Thanks, we have replaced “should” with “could” in our conclusion: “The WHO/UNICEF IMCI protocol could use pulse oximeters for pneumonia case management, instead of making a conditional recommendation for its use.”

18. Please present and organize the Discussion as follows: a short, clear summary of the article's findings; what the study adds to existing research and where and why the results may differ from previous research; strengths and limitations of the study; implications and next steps for research, clinical practice, and/or public policy; one-paragraph conclusion.

Thanks, we believe our discussion section already follows this convention. Please let us know if there are specific aspects of the presentation of the Discussion you would like amending.

19. S1 Appendix table A1- is the date seen relevant? I imagine this is identifying along with the number of episodes, age and whether they were seen by CHW or at HC. Please amend this table. Manuscripts submitted to PLOS should not contain research participants personally-identifying information. In rare exceptions where this is unavoidable and a manuscript does contain PII, the authors should be willing and able to provide PLOS with confirmation of GDPR compliance upon request.

Thanks for pointing this out. We have removed the dates and replaced them with Days since first seen e.g. Day 0, Day 3 etc. This information should not be identifying because the specific location is not stated. We opt to include the Days since first seen as it is useful to understand care seeking patterns for multiple pneumonia episodes in the same children.

20. Map of Malawi- you may  consider adding this into the main text as it gives an immediate visualisation of where the data were  collected from

Thanks, we have added this map to the main paper as Figure 1, referenced in the first paragraph of the Methods section.


Comments from the reviewers:

**Reviewer #1:**

This is a very important piece of work that merits consideration by the journal as it tries to assess the "… mortality outcomes of infants and children diagnosed and referred using clinical guidelines with or without pulse oximetry in Malawi". Recent advances in the wider scale up and implementation of pulse oximetry need to go hand in hand with data like the one presented in this manuscript, which are clear-cut. Pulse oximetry helps identify children at risk of dying. Authors conclude that "Pulse oximetry identified fatal pneumonia episodes at HCs in Malawi that would otherwise have been missed by WHO referral guidelines alone", which is a statement with which I do agree. I believe the journal should consider (if finally accepting the manuscript) the inclusion of a comment to go hand in hand with the manuscript. I only have a few minor comments to add:

We are very glad you recognize the importance of our work, thank you for taking the time to review our paper.

1. *          The word data should be used in plural

We have checked and amended.

2. *          Current global estimates for Pneumonia mortality are closer to 800,000 than to 900,000/year

We have updated and text to reflect 800,000 and changed reference #1 to the following:

1. GBD Lower Respiratory Infections Collaborators. Quantifying risks and interventions that have affected the burden of lower respiratory infections among children younger than 5 years: an analysis for the Global Burden of Disease Study 2017. Lancet 2020;20:60-79*.*

3. *          The fact that mortality as an endpoint was assessed at day 7 but also at day 30 means that many of these deaths may not (or may indeed) be related to the initial episode that took the patient to the health system (particularly for those at day 30). This should be further discussed, but it doesn't eliminate the validity of the association and therefore of the use of oxygen saturation as a risk stratification tool

Although we prespecified our primary end point to be mortality within 30 days, after doing the data linkage all of the deaths within 30 days were actually within 7 days or less, so the issue the reviewer describes is not actually present. We state this in the first paragraph of the results section: “*16 (3.8%) of the 417 CHW episodes and 13 (1.9%) of the 695 HC episodes died within seven days of diagnosis. There were no deaths between eight and 29 days after the initial assessment. A further 9 CHW and 17 HC children were recorded as dying between 30 and 1537 days after outpatient diagnosis and are included in the survival at 30 days groups.*”

4. *          In this respect, is there any information (for example of verbal autopsy results) regarding the potential underlying causes of those deaths? It would be very helpful to be able to state that an elevated proportion of those deaths were secondary to respiratory problems

We were unable to link these data to verbal autopsies being conducted in the district at the time. However, please see our response to point 3 immediately above. All deaths included in our analysis occurred on day 7 or earlier so are likely to be linked to the acute episode of pneumonia. Deaths after day 30 (i.e. not within 30 days) or later were included in the survival to 30 days group for the mortality analysis (i.e. did not have the primary endpoint of mortality within 30 days due to acute pneumonia).

5. *          I understand vaccination data were also collected. Was there any association between mortality and lack of adequate vaccination against Hib or pneumococcus?

This question, while important, was beyond the scope of this study, which is focused on outpatient pulse oximetry and mortality. We have previously published associations between PCV13 and RV1 introduction in this population on mortality.

McCollum et al., 2017: [10.1371/journal.pone.0168209](https://dx.doi.org/10.1371%2Fjournal.pone.0168209)

Bar-Zeev & King et al., 2018: <https://www.sciencedirect.com/science/article/pii/S2214109X18303140>

6. *          The findings that association with mortality is maintained with threshold 93% is also very important but has the risk to overload the (already fragile) system if all patients fulfilling this criterion are recommended for a transfer. Have you been able to conduct any economic modeling of costs associated with the two scenarios (transfer only if sat<90%, or transfer when <93%?). This would be super helpful, as I predict that costs to the system would be massively increased (and be unassumable). Were there other specific variables associated to mortality within the specific group with saturations between 90-93% which were also associated with mortality? Could the recommendation include a need to fulfil at least two of the risk factors, rather than one?

We have not conducted any economic modeling related to these questions. Although we agree that such modeling is important it is beyond the scope of this analysis. We are planning such work as our next paper.

In addition, while we agree that examining whether other covariates were associated with mortality among children with an oxygen saturation between 90-93% may be of interest, this is a different question than what we sought to address. Our a priori plan was to evaluate whether prespecified oxygen saturation criteria were independently associated with elevated risk of mortality among outpatient children with pneumonia. We did not seek to address whether other variables were independently associated with mortality among children with an oxygen saturation of 90-93%. This is a different, and interesting, question that could be addressed in a separate analysis, but in our view would distract from our pre-specified objectives in this paper.

7. *          Nothing is mentioned on the absence/availability of emergency oxygen/systems for transfers. Recognizing this is a major deficit in the health systems of LMICS, it may be worth stating it as an important consideration for the future. There is an ethical dilemma of measuring oxygen saturation but not being able to provide life-saving oxygen, at least for the transport. This is perhaps the most neglected field in pneumonia research, how to ensure cheap and durable availability of oxygen for emergency transport

Authors: Thank you for this suggestion. We have added the following sentence at the conclusion of the discussion section:

“As pulse oximetry use increases in outpatient settings critical next steps include addressing oxygen availability at clinics and during transportation to hospitals.”

8. *          Pulse oximter readings are very variable and often can produce false positive results of hypoxemia values which are not real. I understand that there was a specific training conducted, but perhaps it may be useful to add in the methods section how values obtained in the peripheral health system were considered "reliable" and robust (i.e whether you had to repeat more than once the reading, or you had to ensure the heart rate was also considered credible etc…). This is particularly important with one of your conclusions in relation to the association with mortality: "the failure to obtain an SpO2 measure using pulse oximeters in identifying otherwise unrecognized fatal childhood pneumonia cases accessing primary care". Could some of those cases be failures of the measurement technique? Of the devices used? It may seem very obvious, but this appears important to me.

Authors: Thank you for this comment. We have previously published a detailed report that includes the criteria we used to train healthcare workers to determine whether or not an oxygen saturation measurement was a quality recording and when it was appropriate to finalize the measurement. This is described in the below citation (reference [6] in the paper), which we cite in this manuscript in the methodology section with the subheading for “Pulse oximetry measurements.”

McCollum ED, King C, Deula R, et al. Outpatient pulse oximetry implementation with rural facility and community health workers during three years of child pneumonia care in two central Malawi districts. Bulletin of the World Health Organisation 2016; 94: 893-902.

This original study was not designed to independently assess the quality of all pulse oximetry measurements completed throughout the project period. The project did not have the capacity to do this. Rather, we conducted frequent supportive supervision visits (monthly) with providers to evaluate their pulse oximetry measurement technique on actual pediatric patients in order to address and correct any deviation from measurement procedures. We also conducted a mid-study observation where provider measurements were directly observed in a group setting and remediation was provided, and healthcare provider measurements were compared to a reference pediatrician measurement. All of this is described and reported in detail in the citation provided above, and also alluded to in this write up. Based on these observations and the findings that we are reporting (i.e., failed measurements are independently associated with mortality), it is likely that the vast majority of these failed measurements can be attributed to severe illness in the child (i.e., poor perfusion related to hypovolemic or distributive shock, etc.).

For full clarity we have added the following sentence in the methods section for “Pulse oximetry measurements.”

“Providers were trained to record measurements that demonstrated consistent plethysmography waveforms along with a stable, non-drifting oxygen saturation and age-appropriate pulse rate. Given this work was conducted within a routine clinical context providers were not required to repeat measurements meeting these quality criteria.”

Reviewer #2: PLOS Medicine Colbourn SpO2 Malawi review

The lowest denomination of low-cost factors to determine poor outcome is required. Oxygen saturation monitors will be increasingly available for use in developing health and an assessment of their impact is required. 
This first report is very useful for informing this field and provides structure to the reporting of future studies.

Authors: Thank you

Comments

Methods

1. Describe the process for patients to access and move through the HC system (i.e. HCW, HC, OP referral, Hospital in patients).

Authors: We have added the following sentences to a new “Setting” sub section in the methods section.

“In the Malawi healthcare system children are intended to access care at either village clinics or health centres. At the village clinic level if the child is found to be referral eligible then the child is expected to be referred by the community healthcare worker to either the health centre or hospital. At the health centre level children meeting referral criteria are referred to hospital.”

Results

2. P10 L227. This manuscript would be helped by a figure or initial text in the results that provide cascade detail to the patients, matching and deaths to provide better context to what is reported.

i.e. N = 13814 pneumonia episodes, 6941 CHW + 5761 HC in which SpO2 <90% found in 86/6941 (1.2%) and 608/5761 (10.5%) respectively. There was matching of pneumonia episodes to mortality cohort in N=1112, 417/6941 CHW and 695/5761 HC.  of which there were 29 deaths representing 2.6% of the matched episodes (16/417 CHW and 13/608).

We have included this as Figure 2

3. P10 L252 The case for chest indrawing is well made.

Authors: Thank you

Page 11 L259

3. In light of the finding of a discrepancy in the SpO2 <90% and deaths in CHW cohort (missing 75% of deaths), please provide discussion on the use of an adult probe in a paediatric community setting and the potential for error from extraneous light and the difficulty in positioning. Please also consider discussion that

Authors: We’ve added additional information to the methodology section to provide more details on the how providers applied the probe on the child and the metrics used to determine a final, quality measurement. CHWs providing care at informal clinics were made aware of potential issues with extraneous light and these issues were addressed, and, if needed, corrected during monthly supportive mentorship visits made by the study team. In addition, by applying the probe to the child’s toe (for those <2 years old or <10 kg) there were infrequent issues with positioning in our experience. We’ve added the following to the ‘pulse oximetry measurements’ sub-section in our methods section:

“Healthcare providers underwent a one-day training in pulse oximetry, medical record keeping, and the definition of pneumonia at the start of the study period; they had continued support through monthly mentorship visits. SpO2 measurements were taken using the Lifebox device (Acare Technology, Xinzhuang, Taiwan, China), with a universal adult clip probe applied to the child’s big toe if less than two years of age or below 10 kilograms. Otherwise for older or heavier children providers were instructed to use either the big toe or an appropriately sized finger. CHW and HC workers were trained and re-trained in the use of pulse oximetry by a paediatric pulmonologist (EDM) as described by McCollum et al [6]. Providers were trained to record measurements that demonstrated consistent plethysmography waveforms along with a stable, non-drifting oxygen saturation and age-appropriate pulse rate. Given this work was conducted within a routine clinical context providers were not required to repeat measurements meeting these quality criteria.”

Table 1 

4. The results identify that an SpO2 threshold of 93% would result in a 6 times higher referral rate by CHW and double referral rate by HC. Please discuss the potential impact of this on the ability to deliver safe and effective care to children with pneumonia with increased sensitivity to identify severe disease, but the potential impact of reduced sensitivity having an economic and hospital bed impact that could reduce the ability to priortise services for those with severe disease.

Thanks for raising this issue. Although at CHW level there are approximately 6 times the number of <93% SpO2 cases (n=14+520=534) than <90% (n=7+79=86) episodes, the number of <93% cases is still only around half of those identified clinically by danger signs (n=41+962=1003). Considering there is some overlap with those clinically identified, the SpO2 threshold of <93% used in conjunction with clinical criteria would therefore not actually identify that many more children for referral. At the HC level the numbers identified clinically (n=110+612=722) are fewer than the numbers identified by <93% SpO2 (n=128+1056=1184) so this may be more of an issue. We have added a sentence discussing this point to the third paragraph of our discussion section: “Whilst pulse oximetry in conjunction with clinical signs can identify additional fatal pneumonia episodes than clinical signs alone this increased sensitivity of diagnosis comes at the price of reduced specificity (Table 6), and therefore has potential to overwhelm weak hospital systems via identifying additional cases for referral to hospital. Detailed understanding of local hospital system capacities will be fundamental when deciding the appropriate SpO_2_ threshold for referring patients.”


Discussion

5. Key finding is that study identifies that clinical criteria including chest indrawing together with SpO2 <90% fail to identify 75% of deaths from pneumonia in children. It is difficult to understand that 75% of deaths occurred in children whose parents were concerned enough to attended services so early that they had no severe signs and no desaturation, but then did not re-attend when things got worse. As this represents such a large proportion of 'inaccessible pneumonia deaths' it would be useful to consider how this may be resolved in a future study, i.e red flag information.

Thanks for raising this issue. We already discuss it in the fourth paragraph of our discussion section, mainly in relation to lack of CHW training: “Notably, 12 (75%) of the 16 deaths in CHW episodes that occurred within seven days of diagnosis were neither hypoxemic nor clinically eligible for referral highlighting the importance of follow-up and continuing case management, as well as more specific diagnostic approaches (S3 Appendix pp11–12 has further detail on these episodes). CHWs may also have missed danger signs, meaning support for training, supportive supervision and mentoring for iCCM may be important in this context.”

6. Please discuss that health seeking behaviours are orientated to younger children and it is older children that do not attend out patient services after referral. Whilst this is regrettable, parents so appear to understand that younger children are at greater risk.

Age was not a significant predictor of mortality in our adjusted analyses (S3 Appendix Table A3) and we do not focus on it or present it in our analyses of referral outcomes. We therefore feel that discussing this point is beyond the scope of our results.

Please also note that even in young infants with any sign of infection, referral acceptance rates are low as documented by PSBI IR papers, see below:

Wammanda RD, Adamu SA, Joshua HD, Nisar YB, Qazi SA, Aboubaker S, et al. Implementation of the WHO guideline on treatment of young infants with signs of possible serious bacterial infection when hospital referral is not feasible in rural Zaria, Nigeria: Challenges and solutions. PLoS One. 2020;15(3):e0228718.

Guenther T, Mopiwa G, Nsona H, Qazi S, Makuluni R, Fundani CB, et al. Feasibility of implementing the World Health Organization case management guideline for possible serious bacterial infection among young infants in Ntcheu district, Malawi. PloS one. 2020;15(4):e0229248-e.

7. Overall the study is of value for providing an indication of the potential value of pulse oximetry in childhood pneumonia  - but large missing data and relatively few deaths limit the strength of conclusions despite rigorous analysis. The conclusions could be more circumspect. Line 300 - This evidence 'could' support…  The evidence in itself is not strong enough to support the inclusion at this time. Line 420 'should' is inappropriate based on this evidence alone.

Thanks, we have replaced “should” with “could” in our conclusion: “The WHO/UNICEF IMCI protocol could use pulse oximeters for pneumonia case management, instead of making a conditional recommendation for its use.”

Reviewer #3: Alex McConnachie, Statistical Review

Colbourn et al consider the potential for the use of pulse oximetry for identifying children likely to die from pneumonia in Malawi. This review looks at the use of statistics in the paper.

Unfortunately, I have a number of concerns.

1. A major problem is the very poor linkage rate, which casts doubt on the validity of the results. The authors comment that there were some differences between those matched and not matched, with reference to the tables and appendix, but should perhaps expand on what these differences were in the main text.

Thanks for your review. Whilst we appreciate our linkage rate was low, as our study is the only study we are aware of that links pulse oximetry use at outpatient level to mortality outcomes for children in LMIC we believe it is valuable, as you also state below in your fourth point.

We had removed the material on the differences between those matched and those not matched to the Appendix to shorten the length of our main manuscript. We have now added these paragraphs back into the results section of the main manuscript (paragraphs 2 and 3 of the Results section).

2. My main concern with the analysis is the regression modelling. There were problems with convergence, and the models that it had been intended to fit (allowing for clustering, and including additional covariates and interaction terms) were not possible. There were generally few events, and the models that were fitted produced estimates with very wide confidence intervals. Some models reported in the supplement only worked using a logit link. All of these factors suggest that these models are unstable, and I would not trust these results.

Whilst it is true that there were few death events and the results of the models have wide confidence intervals, we interpret the results in light of this uncertainty. We follow our prespecified analysis plan (now included as Appendix S1) though do now also include the additional sensitivity and specificity analyses you have requested in point 4 below.

3. Note that the description of the model is not entirely correct. On line 184, mu.i is not death for subject i, it is the probability of death; Yi is not the log risk of death, it is the outcome, death, for subject i.

Thank you for pointing out these errors, we have corrected this description of the model and this line now reads: “Where $\mu_{i}$ is the probability of death for individual i, $Y_{1}$ is the outcome, death, for individual *i*,”

4. Nevertheless, there may be valuable data here, but the tables as presented are quite complex, and are not easy to follow. A simpler approach might be to report more standard measures of diagnostic performance, i.e. sensitivity, specificity, PPV, NPV. That might show, in terms that are easily understood, which combinations of screening criteria (WHO, Malawi, SpO2 <90%, or <93%) worked best. I doubt that the sample size is enough to test say whether any differences are statistically significant, but might make the case for additional research.

Thanks, we have now added this analysis as Table 6, and added the following text describing these results to the end of our Results section:

“Table 6 shows the sensitivity and specificity of pulse oximetry with clinical signs versus clinical signs only in identifying deaths. We focus on sensitivity as the objective is to identify as many of the deaths as possible. At CHW level, using both SpO2 thresholds, the sensitivity was 25% compared to a sensitivity of 12.5% for Malawi clinical ICCM criteria only. At HC level, both <90% and <93% SpO2 eligibility thresholds give a sensitivity of 85% compared to a sensitivity of 77% for Malawi clinical IMCI criteria only. At HC level, in scenarios using WHO clinical criteria 9 of the 13 deaths are identified by the <90% SpO2 eligibility threshold and 10 of the 13 deaths are identified by the <93% SpO2 eligibility thresholds. These sensitivities of 69% and 77% compare to a sensitivity of 38% for WHO clinical IMCI criteria only. Given the small numbers of deaths the differences in sensitivity between the scenarios are not statistically significant.”

We have described the methods for this analysis in a new paragraph at the end of the analysis sub-section of the methods: “We calculated the sensitivity and specificity of clinical and SpO2 eligibility on the mortality outcome and compared it to the sensitivity and specificity of clinical eligibility only, for SpO2 eligibility thresholds of <90% and <93% SpO2, WHO and Malawi eligibilities, at CHW and HC levels. We included SpO2 failed measurements, as well as SpO2 below threshold as SpO2 eligible in these analyses, given failed SpO2 measurements are associated with mortality.”

5. Overall, the conclusions of the paper, that SpO2 measurements (or lack of), and chest-indrawing should be included in screening criteria, seems premature given these data.

Thanks, we have replaced “should” with “could” in our conclusion: “The WHO/UNICEF IMCI protocol could use pulse oximeters for pneumonia case management, instead of making a conditional recommendation for its use.”

6. Minor points on Table 1: p values of "0.000" should be reported as "<0.001", and there is no need to add asterisks - the actual p-values are reported.

Thanks, the presentation of the p-values in Table 1, also Tables 3 and 5 have been revised accordingly

7. Table 2 is confusing - some percentages are in columns, some in rows, but as stated above, if the focus were changed to measures of diagnostic performance, that might help/

We have added “col” and “row” to the headings in Table 2 to clarify which are column and which are row percentages. The groups in Table 2 are specified in our pre-specified analysis plan (now included as Appendix S1). We have also included the measures of diagnostic performance in Table 6 as requested.

8. Tables 3 and 5 mix up risks and odds, with the constant term referred to as baseline odds.

Thanks, this has been corrected to baseline risk

9. I note that individual consent was not obtained for this study, but individual patient data is reported in the supplement, which could potentially identify individuals.

Thank you for pointing this out. As per our response to the Editor’s point 19 above, we have removed the dates and replaced them with Days since first seen e.g. Day 0, Day 3 etc. This information should not be identifying because the specific location is not stated. We opt to include the Days since first seen as it is useful to understand care seeking patterns for multiple pneumonia episodes in the same children.

Reviewer #4:

1. page 6 the phrase chest in drawing might likely is the same as retractions, a term more familiar to north american. Please add

Thank you for this comment. For unfamiliar readers we have added a description of chest indrawing that is consistent with the World Health Organization. The following text has been added near the end of the fourth paragraph of the introduction:

“…included mandatory hospital referral for 2-59 month old children with chest indrawing (i.e., bilateral inward pulling of the lower anterior subcostal tissue during inspiration).”

2. page 7 it is customary to add the name and address and even model number of a device used in research. Please add

This has been added (Acare Technology, Xinzhuang, Taiwan, China).

3. Also, please detail why an adult universal probe was used in young children. did it work better than pediatric one or thats all there was? Thank you.

During this time period (the year 2011 when the pulse oximeters were procured) a pediatric probe was not available with this pulse oximeter device. We have added the following language to the methods section:

“SpO2 measurements were taken using the Lifebox device (Acare Technology, Xinzhuang, Taiwan, China), with a universal adult clip probe applied to the child’s big toe if less than two years of age or below 10 kilograms. Otherwise for older or heavier children providers were instructed to use either the big toe or an appropriately sized finger. A pediatric probe was not available during this time period. “

4. Page 10. Please define what SpO2 eligible means

This refers to eligibility for referral based on the child’s oxygen saturation. For readability purposes we do not always repeat “SpO2 eligible for referral” and when used multiple times in a sentence or paragraph we do shorten this to SpO2 eligible. We feel that this should be clear to readers based on how we are using the phrase. This can be changed if the editors agree that the current wording is not understandable.

5. Page 13. Please expand the discussion about specific limitation with SpO2 measure in your specific setting. It's what people would likely want to read in the paper.

This reviewer’s comment is unclear to the authors and will need further clarification in order for us to respond constructively. However, we have previously published qualitative data on the opportunities and barriers of pulse oximetry use in this setting.

King et al., 2018: <http://dx.doi.org/10.1136/bmjopen-2017-019177>
