# Supplementary material for: Predictive value of pulse oximetry for mortality in infants and children presenting to primary care with clinical pneumonia in rural Malawi: A data linkage study
Source: PLoS Med. 2020 Oct 23;17(10):e1003300. doi: 10.1371/journal.pmed.1003300 (PMC7584207; doi:10.1371/journal.pmed.1003300)
Supplement: Attachment — Submitted filename: PMEDICINE-D-20-00470R2 Response to review.docx [file pmed.1003300.s005.docx]

**PMEDICINE-D-20-00470R2**

**Predictive value of pulse oximetry for mortality in infants and children presenting to primary care with clinical pneumonia in rural Malawi: a data linkage study**

**Response to Reviewers**

**Requests from the editors:**
  
As you will see, the statistical referee raises significant issues and advises us to reject the manuscript. We have discussed this and feel that we would like to offer another revision opportunity. There is a sense that the presentation is simply trying to stretch limited data too far in producing clinical recommendations - for example, under "what do these findings mean?" all three points are "should be used" or similar, and it seems that "our findings suggest that pulse oximetry could be beneficial... and should be further investigated", say, would be more sensible.

Thank you for your careful review and the opportunity to revise our manuscript. We have taken this point on board and have rewritten the “what do these findings mean? Section of our Author Summary to say:

- Our findings suggest that pulse oximetry could be beneficial in supplementing clinical signs to identify children with pneumonia at high risk of mortality in the outpatient setting in health centres for referral to a hospital for appropriate management.
- In high mortality settings in low and middle income countries, in the absence of pulse oximetry, presence of chest indrawing could potentially be explored as a referral sign to a hospital but needs further research in routine settings.

In addition, please do address all of the points from ref 3 (as well as from other refs). Ref 3 is our statistician and we see these points as important. I realise in some instances toning down will be a compromise.

Thank you, please see our responses below and revised and updated paper with tracked changes.

**Comments from the reviewers:**

**Reviewer #1**: all my comments have been addressed, and I'm pleased how authors have rewritten the manuscript. I'm also happy how they have dealt with other reviewer's comments. I still think, however, that this manuscript would benefit from an accompanying commentary.

Thank you very much for your review, we are glad you are happy with our revised manuscript. We would be very happy if the editor agreed to commission an accompanying commentary.

**Reviewer #2**: Thank you for responding to the primary review. The responses were very helpful and provide clarity.

I have one remaining comment. Whilst I note that the authors have provided detail on the training for CHW in pulse oximetry, that '12 (75% of the 16 deaths) 'CHW may have missed danger signs, meaning further support for training...' could be inferred that CHW may somehow be considered responsible for these omissions as the children were not 'hypoxemic'. 

I would ask that the authors also acknowledge within their limitations, that the use of adult probes on pulse oximeters was a limitation and some of these children may have been hypoxemic at the time of review - we would not use adult probes where a paediatric probe is available - as is increasingly the case globally. The use of adult probes for young children have documented limitations. This potential technological failure to detect hypoxemia does not detract from their core message - but to me adds to it - as it implies that the use of age appropriate sensors may likely provide even greater sensitivity to detect children who may die from lower respiratory tract infection. 

Thank you very much for your review. We agree with your comment on adult probes and have added the following two sentences to our discussion section after the sentence you highlight above to reflect this:

“The use of adult probes for pulse oximetry in our study may have contributed to missed hypoxemic cases. The increasing availability of paediatric probes may provide greater sensitivity to detect children who may die of pneumonia”

**Reviewer #3:** Alex McConnachie, Statistical Review
 
I thank Colbourn and colleagues for their responses to my original comments. 
 
Seeing Table 6 (sensitivity etc.) helps me to understand the data a little better. Pulse oximetry adds sensitivity, at the expense of a loss of specificity. The results section of the paper concentrates on sensitivity, but the loss of specificity it is touched on in the discussion. No confidence intervals are provided for any of the diagnostic measures, but the authors do note that none of the differences are statistically significant.

The diagnostic odds ratios in table 6 are:

A1: 1.26, A2: 1.17, A3: 1.32
B1: 7.13, B2: 6.19, B3: 5.24
B4: 6.57, B5: 6.82, B6: 3.43

This suggests that pulse oximetry may be of no real value in the community setting, but might be in a HC setting, particularly when added to the WHO clinical criteria. It also suggests that the Malawi danger signs may perform better than the WHO danger signs on their own.

Thank you for your careful review and for your suggestion to use diagnostic odds ratios, which we have now added to Table 6. We agree and have now revised our manuscript to de-emphasise the CHW results and tone down the language in our recommendations and conclusions. The last sentence of our abstract is now:

“Our findings suggest that pulse oximetry could be beneficial in supplementing clinical signs to identify children with pneumonia at high risk of mortality in the outpatient setting in health centres for referral to a hospital for appropriate management.”

And the “what do these findings mean? Section of our Author Summary is now:

- Our findings suggest that pulse oximetry could be beneficial in supplementing clinical signs to identify children with pneumonia at high risk of mortality in the outpatient setting in health centres for referral to a hospital for appropriate management.
- In high mortality settings in low and middle income countries, in the absence of pulse oximetry, presence of chest indrawing could potentially be explored as a referral sign to a hospital but needs further research in routine settings.

The diagnostic odds ratio results are added to the end of the results section (please also see tracked change to methods briefly describing the use of DOR and referencing a paper detailing the method):

“The DOR show that at CHW level neither pulse oximetry with clinical signs (Table 6 A1: <90% SpO2 threshold: DOR: 1.26 (95%CI: 0.40, 4.00), A2: DOR <93% SpO_2_ threshold: DOR: 1.17 (95%CI: 0.37, 3.71)) or iCCM clinical signs alone (A3: DOR: 1.33 (95%CI: 0.29, 6.05)) accurately identified those who died. At HC level pulse oximetry was able to accurately identify those who die with both Malawi (B1: <90% SpO_2_ threshold: DOR: 7.13 (95%CI: 1.57, 32.4), B2: DOR <93% SpO2 threshold: DOR: 6.19 (95%CI: 1.36, 28.1)) and WHO (B4: <90% SpO_2_ threshold: DOR: 6.57 (95%CI: 2.00, 21.6), B5: DOR <93% SpO2 threshold: DOR: 6.82 (95%CI: 1.86, 25.0)) IMCI clinical signs. Although the point estimates for these DOR are higher than those for when Malawi and WHO IMCI clinical signs alone are used without pulse oximetry (B3: Malawi: DOR: 5.25 (95%CI: 1.43, 19.2), B6: WHO: DOR: 3.43 (95%CI: 1.10, 10.7)), the confidence intervals are wide and overlapping indicating these differences are not statistically significant.”

We have also revised the first, second and last paragraphs of our discussion section accordingly – please see tracked changes.

These data are limited though, because they are derived from real world data in which children are being assessed and treated. The aim of the assessments and subsequent treatment and referrals will be aiming to prevent adverse outcomes. It cannot be known how many of these children might have died in the absence of treatment. So, the actual number of true positives will be higher, and the number of false negatives lower, than suggested by simply looking at death as the outcome. Using referral and hospitalisation data as in Tables 4 and 5 does not really help, because these decisions are being made in the light of the assessments being done; in this study, there is no "gold standard" against which to assess the alternative screening criteria.

Thank you for raising this issue. We agree that the real-world nature of the data means that there is no real “gold standard” against which to assess the screening criteria. However, the purpose of our analysis is to determine which cases, under real world conditions, can be identified before they go on to die for the purposes of assuring that patients who are at higher risk of dying are actually referred to the hospital for potential life-saving treatment. Though it is true that there is no way to understand what proportion of these children will survive their acute illness, even if all high risk children are referred to a hospital. Therefore, we believe our analysis is relevant for real world case management of children with pneumonia.

I still feel the logistic regression models are pushing the data too far. For death as the outcome, there are only 13 or 16 events being analysed, depending on the dataset, and models are being fitted (or are being attempted) with a total of 6 parameters (intercept, SpO2 (2 df), clinical danger signs, interaction (2 df)), so the events-per-variable ratio is very low. Some of the interaction terms are not estimable due to the lack of events in some subgroups.

We agree that there are limited death events given the number of parameters fitted in these models. However, we acknowledge this as a limitation and clearly show the confidence intervals of the coefficients and where coefficients (for the interaction terms) are not able to be estimated due to lack of events in subgroups. Our latest revisions tone down the significance of our findings, provide additional discussion, and highlight that further investigations are needed. In conjunction with the added diagnostic odds ratio results (see above response) we hope you agree that our manuscript as a whole is appropriate.

When looking at referral and hospitalisation as the outcome, there are more events, but still very few in some subgroups. The text of the paper mentions associations with low SpO2 and with clinical assessments, but these are the main effects, and each applies only in the absence of the other. The interaction terms are not taken into account, and these are generally below 1.

Thank you for pointing this out. The interaction terms are not significant for the CHW referral models (Table 5A), though are, and are below one, for some of the HC referral models (Table 5B). In our results section describing the referral results in Table 5 we do not quote the numerical results of these analysis though we have added the following sentences to the narrative description of these results in the penultimate paragraph of our result section to explain this:

“The interaction terms between the SpO_2_ and danger signs parameters were below one and statistically significant for the following HC referral models (Table 5B): M90 and M93: Outpatient referral decision indication outcome: SpO_2_ <90% X Danger signs; Hospitalization outcome: failed SpO_2_ X Danger signs; and W90: Outpatient referral decision indication outcome: SpO_2_ <90% X Danger signs. These results indicate that the association between the SpO_2_ parameter and the outcome was attenuated by the presence of danger signs.”

I think these data are interesting, and suggest that chest in-drawing and SpO2 measurements may be of value in some settings, but it is quite messy data, which was not collected with this analysis in mind.

The linkage may be highly novel, but the success rate was very low, with many differences between those linked and not linked.

Thank you for your review. We agree the data does have limitations. We believe our paper is still useful for case management of childhood pneumonia in low income settings and, given our latest revisions, hope you agree.
