# Supplementary material for: Predictive value of pulse oximetry for mortality in infants and children presenting to primary care with clinical pneumonia in rural Malawi: A data linkage study
Source: PLoS Med. 2020 Oct 23;17(10):e1003300. doi: 10.1371/journal.pmed.1003300 (PMC7584207; doi:10.1371/journal.pmed.1003300)
Supplement: Attachment — Submitted filename: response to editorial and production requirements.docx [file pmed.1003300.s006.docx]

**PMEDICINE-D-20-00470R4**

**Predictive value of pulse oximetry for mortality in infants and children presenting to primary care with clinical pneumonia in rural Malawi: a data linkage study**

Please see below for how we have dealt with the four remaining queries from publication assistant Jessica Hyde in her emails dated 31^st^ August and 28th August. Below that is the longer list of editorial and production requirements, which we have also met. Thank you.

31^st^ August

1. Please ensure that all Supporting Information files use the following format verbatim throughout the manuscript (including legends and all in-text citations): S1 Fig, S1 Table, S1 Text, S1 Data, etc. Each file type should start at 1 and increase numerically. Please note, however, that the file names themselves must use an underscore rather than a space, i.e. S1_Fig.tif, S1_Table.xlsx, S1_Text.doc, S1_Data.xlsx, S1_PRISMA_Checklist.docx, S1_Appendix.docx, S2_Appendix.docx, etc. Please change your current supporting information file names to the more simplified file names shown here.

We have now simplified the file names of S1 S2 and S3 supporting information files.

2. Please note that your submission must refer to specific files using our naming convention (S1 Appendix, S2 Appendix, S1 Checklist, etc.) Please all update in-text citations and Supporting Information file legends/captions accordingly.

We have now updated the supporting information file names in the legend after the end references section of the manuscript accordingly with the simplified file names.

28^th^ August

Can you please address the following issues as soon as possible? Your paper has been sent back to enable you to make these changes. 

1. We have noted that you have the following present in your manuscript: List of keywords

We have removed these section from your manuscript file as this information is automatically pulled from the submission form. Please make sure that the information in the submission form is correct.

Thank you. I have also deleted the list of keywords from the manuscript now uploaded. We are unable to see any information on keywords in the submission form on the editorial manager system however so are not able to check this information.

2. Please ensure that all Supporting Information files use the following format verbatim throughout the manuscript (including legends and all in-text citations): S1 Fig, S1 Table, S1 Text, S1 Data, etc. Each file type should start at 1 and increase numerically. Please note, however, that the file names themselves must use an underscore rather than a space, i.e. S1_Fig.tif, S1_Table.xlsx, S1_Text.doc, S1_Data.xlsx, S1_PRISMA_Checklist.docx, etc.

We have added underscores to the file names of all Supporting Information files. As explained in our 24^th^ August response below the three Supporting Information files are numbered S1 S2 S3 because they are all of the same file type (word documents). They are all cited in the manuscript correctly.

3. Please ensure that all main figures are referenced in the manuscript as Fig 1, Fig 2, etc. (including capitalization), rather than Figure 1, Figure 2, etc. Please note, however, that the file names themselves must not include the space, i.e. Fig1.tif, Fig2.tif. Please ensure that all main figure files are clearly labeled as such in the File Inventory.

We have just checked this and we comply with these requirements already in our 24th August version as far as we are aware.

4. Please note that your submission must refer to specific files using our naming convention (S1 Fig, S1 Table, S1 Text, S1 Data, S1 Appendix, S1 Checklist, etc.) Please all update in-text citations and Supporting Information file legends/captions accordingly.

We have checked this again and we comply with these requirements already as far as we are aware. Please also see our response to point 2. above. Thank you.

**PMEDICINE-D-20-00470R3**

**Predictive value of pulse oximetry for mortality in infants and children presenting to primary care with clinical pneumonia in rural Malawi: a data linkage study**

Thank you very much for reviewing our paper again, we are very pleased you are planning to accept it. We have complied with the editorial request and all production issues as detailed below in blue

**Requests from Editors:**

Data – the data statement is now fine, but please remove “We can make available the matched/unmatched data actually used for the analysis (just the relevant variables, with personal identifiable information removed) and could upload that to a free to access public repository such as the one held by the corresponding authors university UCL. Please let us know if this is acceptable.”

Thank you, we have removed this text.

Production Issues:

FIGURES and GRAPHICS:

1. Please ensure that all main figures are referenced in the manuscript as Fig 1, Fig 2, etc. (including capitalization), rather than Figure 1, Figure 2, etc. Please note, however, that the file names themselves must not include the space, i.e. Fig1.tif, Fig2.tif. Please ensure that all main figure files are clearly labeled as such in the File Inventory.

We have edited the references to the figures in the manuscript accordingly. The file names already meet the requirements.

TABLES:

1. Please cite main tables in ascending numerical order in your main article file.

We have checked and the tables are already cited in numerical order (on first citation) in the main article file.

METADATA:

1. Please ensure your short title is correct in the submission form, including symbols, italicisation etc. Your short title will be typeset from the submission form directly.

We have checked this and it is correct (“Predictive value of pulse oximetry for mortality in children with pneumonia treated as outpatients Malawi”)

2. In accordance with PLOS' data policy, please ensure that your Data Availability Statement in the submission form clearly identifies how readers can access your data. Note that it will be typeset as it is written so please ensure it is in complete sentences and appears as you would like it to in the published version.

For details on our data policy, please see <https://eur01.safelinks.protection.outlook.com/?url=http%3A%2F%2Fjournals.plos.org%2Fplosmedicine%2Fs%2Fdata-availability&amp;data=02%7C01%7C%7Cf2a6e565714d44d6e01c08d8444efab1%7C1faf88fea9984c5b93c9210a11d9a5c2%7C0%7C0%7C637334453295125188&amp;sdata=uVFIIN65VIX95NjEepH6pPgLV4Y8DsEftgGqO4ddHd0%3D&amp;reserved=0>

Thank you, we have checked this and it is corrected. Please note we have edited it as per the editorial request above.

ARTICLE FILE:

1. Please indicate the corresponding author on the title page with an asterisk (*) and include the corresponding author's email address. Only an asterisk and the email address itself are required.

We have made this change

2. Please remove author degree titles from the byline of your manuscript file.

These have been removed

3. Please ensure that all authors have at least one affiliation listed on the title page and that all listed affiliations are attributed to at least one author. Affiliations should be listed in ascending numerical order in the affiliation list and should match what is provided in the Authors Information in the submission form.

Affiliations should include an institution, a city, and a country (no postcode). US affiliations need the state as well, written out in full (with no abbreviations).

Thank you, we have checked all affiliations and they already conform to these requirements in the manuscript file and are identical in the submission form.

4. Please ensure that all author names are spelled correctly in the manuscript file and the submission form, including the use of a middle name/initials, and any special characters (accents, umlauts, etc.). Please place first/given names before last/family names. We ask that you ensure they match verbatim. Please be aware that correct spelling and formatting of author names in the article file is vital for accurate indexing in PubMed.

Thank you, we’ve checked all author names and they are spelt correctly and are the same in the manuscript file and the submission system.

5. Please ensure that your Ethics Statement is available in the Methods section of your manuscript in its entirety.

Thank you, our full Ethics Statement is already available in the Methods section of our manuscript in its entirety.

6. We have noted that you have the following present in your manuscript: Abbreviations list

We have removed these section from your manuscript file as this information is automatically pulled from the submission form. Please make sure that the information in the submission form is correct.

Thank you, though having checked both the “Additional Information” and “Manuscript data” tabs we can’t actually see a section containing the list of abbreviations on the submission system.

7. You currently have references stating '[data] not shown' on page 8. Please note that PLOS does not permit references to “data not shown.” Authors should provide the relevant data within the manuscript, the Supporting Information files, or in a public repository. If the data are not a core part of the research study being presented, we ask that authors remove any references to these data.

This “not shown” statement on page 8 is not referring to data. It is referring to a numerical value of a term in the regression Equation at the end of page 7. We do have data and use it for this value of this regression coefficient but chose to simplify the representation of the equation as the additional complexity is not required to convey the substance of the equation and would make it harder to read.

REFERENCES:

1. Please make sure all references are cited in ascending numerical order in the text.

Thank you. We believe this is the case as have used EndNote reference management software for all citations so they are automatically cited in ascending numerical order.

SUPPORTING INFORMATION:

1. Please ensure your supporting information files are individually uploaded to the File Inventory as file type 'Supporting Information' and are not present in the manuscript file.

All three supporting information files are separate and not included in the main manuscript file.

2. Please upload your Supporting Information items individually, rather than in a single file, as you refer to them individually in the text. If you would like to retain the single file please rename internal items (e.g. tables) as A, B, C etc. Please also provide a legend for each under the heading Supporting Information. Supporting Information files should be less than 10 MB each. Please edit any citation of internal items within your Supporting Information files to using letters rather than numbers, e.g. “Table A in S1 Text”. Please ensure these changes are made throughout the manuscript and within the Supporting Information files themselves.

Thank you. The there Supporting Information files are individually uploaded. Only Supporting Information file S3 has tables referred to in the main text and we have renamed them to be A and B (A1 becomes A, and A2 becomes B) both in file S3 and in the manuscript. Please note that table B has two sections that are now named I and II but these sections are only referred to in supporting information file S3 and not in the main manuscript.

3. Please ensure that all Supporting Information files are clearly labeled in the file inventory. i.e. S1_Fig.tif, S1_Table.xlsx, S1_Text.doc, S1_Data.xlsx, S1_STROBE_Checklist.docx, etc.

Each file type should be numbered starting at 1. Please also make sure that the file description matches the file name (e.g. S1_Fig.tif is listed as S1 Fig).

Thank you, all three supporting information files are clearly labelled. They are named S1, S2 and S3 because they are all .docx files.

4. Please ensure that each supporting information file has a legend at the end of the manuscript file, after the Reference list.

Thank you, we have added this legend after the reference list in our manuscript.

5. Please note that your submission cannot contain any references to generic "supporting information" files and instead must refer to specific files using our naming convention (S1 Fig, S1 Table, S1 Text, S1 Data, etc.)

Thank you, all supporting information files are specifically referred to in the manuscript.
